# Therapeutic Options Against the New Coronavirus: Updated Clinical and Laboratory Evidences

**DOI:** 10.3389/fmed.2020.00546

**Published:** 2020-09-15

**Authors:** Amélia Carolina Lopes Fernandes, Adson José Martins Vale, Fausto Pierdoná Guzen, Francisco Irochima Pinheiro, Ricardo Ney Cobucci, Eduardo Pereira de Azevedo

**Affiliations:** ^1^Nurse Department, Nurse School, Universidade do Estado do Rio Grande do Norte (UERN), Mossoró, Brazil; ^2^Tocogynecology Department, Medical School, Universidade Federal do Rio Grande do Norte (UFRN), Natal, Brazil; ^3^Graduate Program of Biotechnology, Laureate International Universities - Universidade Potiguar (UnP), Natal, Brazil; ^4^Medical School, Laureate International Universities - Universidade Potiguar (UnP), Natal, Brazil

**Keywords:** COVID-19, SARS-CoV-2, coronavirus, drug treatment, prophylaxis, viral infection

## Abstract

The pandemic caused by the new coronavirus (SARS-Cov-2) has encouraged numerous *in vitro* studies and clinical trials around the world, with research groups testing existing drugs, novel drug candidates and vaccines that can prevent or treat infection caused by this virus. The urgency for an effective therapy is justified by the easy and fast viral transmission and the high number of patients with severe respiratory distress syndrome who have increasingly occupied intensive care hospital beds, leading to a collapse in health systems in several countries. However, to date, there is no sufficient evidence of the effectiveness of any researched therapy. The off-label or compassionate use of some drugs by health professionals is a reality in all continents, whose permission by regulatory agencies has been based on the results of some clinical trials. In order to guide decision-making for the treatment of COVID-19, this review aims to present studies and guidelines on the main therapies that have been and are currently being tested against SARS-CoV-2 and to critically analyze the reported evidences.

## Introduction

The new coronavirus (SARS-CoV-2) is an RNA virus that belongs to the Coronaviridae family and to the Nidovirales order. It belongs to the same beta subgroup of viruses that caused severe acute respiratory syndrome coronavirus (SARS-CoV) and Middle East respiratory syndrome coronavirus (MERS-CoV) in the past decades, sharing 80% and 50% of their genome, respectively (1). Since the first cases of the novel coronavirus disease (COVID-19) reported in Wuhan (China) in late 2019, more than 80,000 cases have been reported in China alone. According to the World Health Organization (WHO), the epidemic COVID-19 peaked between late January and early February 2020 in China and the rate of new cases declined substantially in early March (2). A World Health Organization report published in March estimated a global mortality rate of 3.4% (3) and until July 1, 2020, 10,357,662 confirmed cases were registered, with 508,055 deaths (4).

In most of the cases, the disease caused by SARS-CoV-2 is represented by a mild upper airway infection with symptoms that include fever, cough, sore throat, shortness of breath, loss of smell and taste, as well as diarrhea (5). However, it can progress to severe acute respiratory syndrome in a short period of time. The virus usually infects the type 2 alveolar cells in the lung, which may explain the severe alveolar damage found in cases of SARS-CoV-2. Due to the astonishing number of COVID-19 patients requiring hospitalization and mechanical ventilation, this current pandemic has already strained healthcare systems in several countries (6).

To date, multiple therapies have been proposed based mostly on the findings of *in vitro* studies and on observational and clinical trials. In these studies, researchers investigated the efficacy and safety of new and old drugs by studying their potential in inhibiting the entry and fusion of the virus within the cells, in controlling viral replication, in suppressing the intense inflammatory response and in controlling hypercoagulability (6–8). In a recent review, Sanders et al. presented a panel of articles published in English that focused on the treatment of adults with COVID-19. The authors admitted that the growing number of publications on therapies against this virus indicates that discoveries about such therapies are constantly evolving (9).

Although no effective vaccine or drug has been approved to treat COVID-19 until the date of writing this paper, some clinical trials have been carried out with already approved drugs, as well as with vitamins and biological samples with promising effectiveness. The aim of this work is to review the literature about which therapies are being researched against the new coronavirus, update the data published in previous reviews and critically evaluate the evidence from the *in vitro* and *in vivo* studies.

## Method

For this review, the inclusion criteria were guidelines as well as clinical, *in vivo* and *in vitro* studies that investigated the use of drugs, chemicals, vitamins and biological agents, with reported efficacy and adverse effects, intended for COVID-19 prophylactic and/or therapeutic purposes.

Guidelines and articles published until July 20th, 2020 were searched without language restriction in Pubmed, Embase, Scopus, and Up ToDate databases. Search terms included coronavirus, severe acute respiratory syndrome coronavirus 2, 2019-nCoV, SARS-CoV-2, COVID-19 in combination with therapeutics, therapy, treatment, *in vitro*, drug evaluation studies, cohort studies, clinical trials, guidelines and pharmacology. The search resulted in a total of 3,948 articles. The authors independently reviewed the titles and abstracts for inclusion. Additional relevant articles were identified based on the citations and references of each paper.

The drugs are presented in sections arranged in alphabetical order, with the critical analysis of the evidences being individually presented. A summary of the selected studies, researched drugs and dosage regimen is presented in Table 1.

**Table 1:** Summary of the drugs and vitamins that have been investigated for prophylaxis and treatment of COVID-19 infection with their respective recommended dose and posology.

**Author (Reference)**	**Study design**	**Drugs**	**Dose and posology**
Yao et al. (10)	*In*-*vitro*	Hydroxychloroquine and chloroquine	Hydroxychloroquine – 400 mg, twice daily, followed by 200 mg twice daily for 4 days Chloroquine – 500 mg twice daily 5 days
Huang et al. (11)	Randomized clinical trial	Chloroquine, lopinavir, and ritonavir	Chloroquine – 500 mg twice daily 10 days. Lopinavir/Ritonavir 400/100 mg, twice daily, for 10 days
Gautret et al. (12)	Open label non-randomized clinical trial	Hydroxychloroquine and azithromycin	Hydroxychloroquine – 600 mg daily, followed by 200 mg twice daily for 10 days Azithromycin – 500 mg on day one, followed by 250 mg per day for 04 days
Lagier et al. (13)	Cross-sectional	Hydroxychloroquine and azithromycin	Hydroxychloroquine – 200 mg three times daily for 10 days Azithromycin – 500 mg on day one, followed by 250 mg per day for 4 days
Mitjá et al. (14)	Randomized clinical trial	Hydroxychloroquine	Hydroxychloroquine – 800 mg on day1, followed by 400 mg once daily for 6 days
Skipper et al. (15)	Randomized clinical trial	Hydroxychloroquine	Hydroxychloroquine – 800 mg on day1, followed by 600 mg once daily for 5 days
Cavalcanti et al. (16)	Randomized clinical trial	Hydroxychloroquine and Azithromycin	Hydroxychloroquine at a dose of 400 mg twice daily plus azithromycin at a dose of 500 mg once daily for 7 days
Borba et al. (17)	Randomized clinical trial	Chloroquine	Chloroquine – 600 mg twice daily for 10 days Chloroquine – 450 mg for 5 days, twice daily only on the first day
Tang et al. (18)	Cross-sectional	Enoxaparin	Enoxaparin– 40–60 mg per day for at least 7 days
Duan et al. (19)	Cross-sectional	Convalescent plasma	Convalescent plasma– 200 ml single dose
Health Alert Network (20)	Guidelines	Interferon-alpha (IFN-α); lopinavir/ritonavir	Interferon-alpha (IFN-α) in 5.000U twice a day (bis in die – BID); Lopinavir/ritonavir (400/100mg twice a day through oral route)
Wang et al. (21)	Cohort	Favipiravir + oseltamivir	Favipiravir 1,600 mg BD on day 1 and 800 mg BD on 2–10 days + Oseltamivir 75 mg BD once a day for 10 days
Goldman et al. (22)	Randomized	Remdesivir	Remdesivir 200 mg intravenous on day 1 and 100 mg for 9 days Or Remdesivir 200 mg intravenous on day 1 and 100 mg for 5 days
Wang et al. (23)	Randomized double-blind Controlled Multicentric Trial	Remdesivir	Remdesivir 200 mg intravenous on day 1 and 100 mg for 9 days
Chen et al. (24)	Randomized clinical trial	Oseltamivir Ganciclovir Lopinavir/ritonavir	Oseltamivir 75 mg twice a day through oral route Ganciclovir 0.25 mg twice a day intravenous Lopinavir/ritonavir 500mg twice a day, oral route
Caly et al. (25)	*In vitro* controlled trial	Ivermectin	5 μM No correlation with human dose
Rossignol (26)	Clinical trial	Nitazoxanide + Hydroxychloroquine; Hydroxychloroquine	Nitazoxanide 500 mg + Hydroxychloroquine 200 mg twice a day for 10 days; Hydroxychloroquine 200 mg twice a day for 10 days
Grant et al. (27)	Review based on several clinical trials	Vitamin D	Daily dose of 10,000 IU of vitamin D3 for a few weeks and once the levels of 25(OH)D increases, the daily dose should decrease to 5,000 IU

## Drugs and Perspectives

### Anticoagulants

Recent studies (11, 24) have demonstrated that patients infected with SARS-CoV-2 who have progressed to viral pneumonia with severe respiratory distress syndrome were diagnosed with disseminated intravascular coagulation and presented abnormal coagulation results during the later stages of the disease. In those infected, increased concentrations of D-dimer and other fibrin degradation products were associated with poor prognosis. Fibrinolysis and pulmonary coagulation are believed to be regulated by several pro-inflammatory cytokines, however, the concrete mechanisms for coagulopathy have not been identified yet (28, 29).

**Figure 1:**
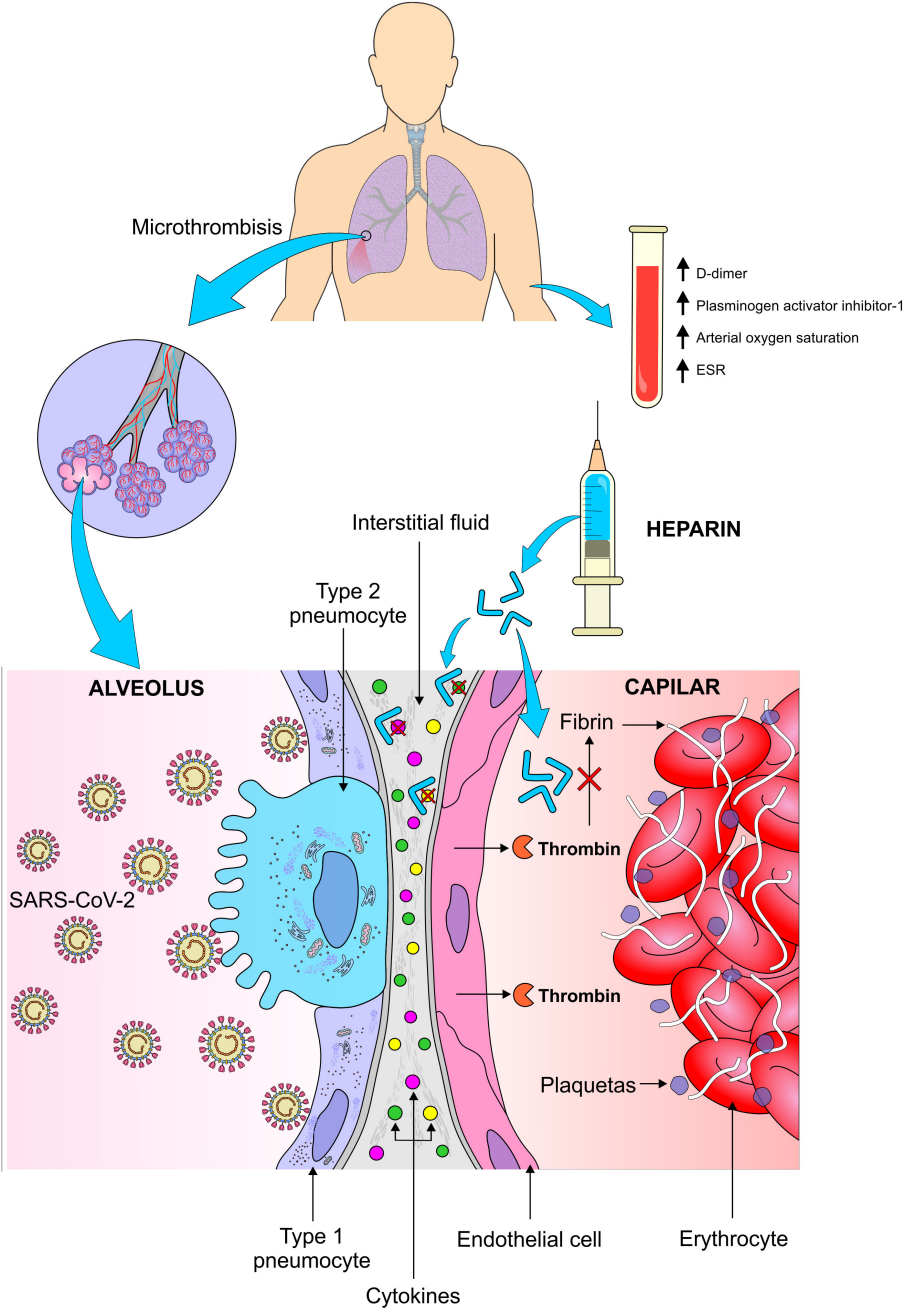
Thromboembolic complications in patients with pulmonary infection caused by SARS-CoV-2 and the mechanism of action of heparin in the pulmonary microthrombotic events. This viral infection results in high levels of cytokines in the pulmonary interstitial fluid, in addition to increased production of thrombin by the pulmonary endothelium, which increases the thromboembolic events in the lung tissue resulting in less oxygenation. The use of heparin reduces the conversion of thrombin to fibrin and decreases the activity of cytokines in the pulmonary interstitium. TSE, Erythematous sedimentation rate. *Figure source: Authors' own drawing*.

Infection-induced endothelial cell dysfunction results in increased production of thrombin which might lead to a state of hypercoagulability. In addition, hypoxia resulting from severe viral pneumonia can stimulate thrombosis due to increased blood viscosity (28). Ozolina et al. (30) demonstrated that the plasma concentrations of tissue factor and plasminogen activator inhibitor-1 were significantly higher in patients with severe respiratory distress syndrome (SARS) in comparison with those without the syndrome (Figure 1).

A recent review reported studies that compared the D-dimer and fibrin values among patients with COVID-19. The authors point out that coagulopathy seems to be related to the severity of illness and to the extension of the inflammatory process and not to the intrinsic viral activity. In addition, they stated that elevated D-dimer at hospital admission has been associated with increased mortality (31).

A meta-analysis (32) including 9 randomized controlled trials and 465 patients with SARS showed that among those treated with low molecular weight heparin, a significant reduction in mortality was observed. Thus, Chinese clinicians decided to use anticoagulants in patients with SARS-CoV-2 as they believed that such drugs could significantly reduce mortality. Seffer et al. (33) claim that viruses bind to immobilized heparin in a similar way as heparan sulfate interacts with the cell surface. This binding is non-reversible and as such, the pathogens are removed from the bloodstream. Thus, they conclude that since heparin has already shown to be effective in reducing viral load in animal models infected by Zica virus, cytomegalovirus, adenovirus and SARS-CoV-2, additional clinical trials are need in order to prove its effectiveness in the treatment of patients with COVID-19.

Tang et al. (18) made a retrospective survey of 449 patients (268 men) aged ≥18 that were hospitalized in China due to serious respiratory problems (respiratory rate > 30/min, arterial oxygen saturation ≤ 93% at rest and PaO_2_/FiO_2_ ≤ 300 mmHg) as a result of COVID-19. Of these, 99 received heparin, mainly low molecular weight (94 received enoxaparin. 40–60 mg per day) for at least 7 days. According to the authors, the early use of anticoagulant therapy in severe cases of COVID-19 was suggested in China based on the analogy with what is known to occur in other viral infections. This hypothesis has been corroborated by a recent evidence of occlusion necropsy and formation of microthrombi in small pulmonary vessels in those infected with the new coronavirus. This study demonstrated that 28-day mortality was no different between heparin users and non-users (30.3 and 29.7%, respectively), but was significantly lower (40.0 vs. 64.2%, *p* = 0.029) in the group of heparin users with severe coagulopathy induced by sepsis and also in those with D-dimer > 6 times the upper limit of normal (32.8 vs. 52.4%, *p* = 0.017). The authors suggest that only patients with more severe forms of COVID-19 (those with considerably high D-dimer) may benefit from anticoagulant treatment, especially with low molecular weight heparin (Figure 1). However, they claim that such findings need to be confirmed by prospective studies.

Although the study of Tang et al. is the only one so far that investigated the use of anticoagulant therapy in patients infected with SARS-CoV-2, the International Society of Thrombosis and Haemostasis (ISTH) recently published a protocol on the management of coagulopathy. In this document, the recommendation is that treatment with low molecular weight heparin (enoxaparin 40–60 mg/day) should be considered for all patients infected with the new coronavirus receiving hospital care, even the patients with mild infection, except for those with active bleeding and platelets below 25,000. The authors stated that there is an additional benefit for using low molecular weight heparin due to its anti-inflammatory properties, which might contribute to a reduction of pro-inflammatory cytokines and may prevent the disseminated intravascular coagulation, which is so common in patients with sepsis (34).

### Anti-inflammatories

**Figure 2:**
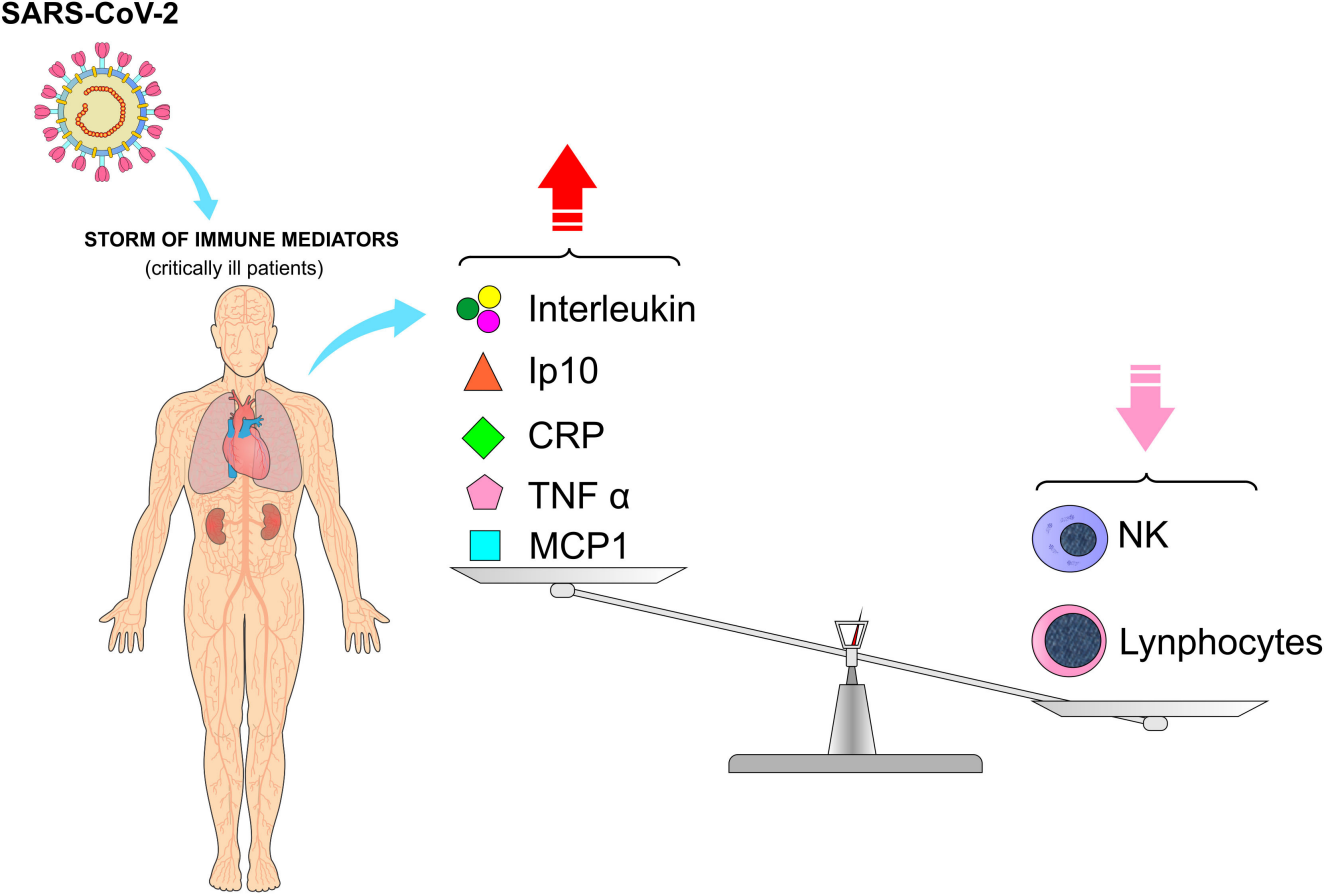
Cytokine release syndrome (CRS) and low levels of lymphocytes in peripheral blood, especially natural Killer cells (NK), in patients infected with SARS-CoV-2. The storm of immune mediators due to infection by SARS-CoV-2 has a direct impact on laboratory tests, with an increase in interleukin, Ip10, CRP, TNT-α, and MCP-1 and a decrease in some of the blood elements such as lymphocytes and NK cells. (Ip10, induced protein 10; CRP, C-reactive protein; TNF-α, tumor necrosis factor; MCP-1, monocyte chemoattracting protein 1). *Figure source: Authors' own drawing*.

Immune mediators such as inflammatory cytokines and chemokines, including interleukins (IL-1β; IL-6, IL-7, IL-8, IL-9, IL-10), induced protein 10 (IP10), C-reactive protein, tumor necrosis factor (TNF-α), monocyte chemoattracting protein 1 (MCP-1), are significantly elevated in patients with COVID-19. The presence of these mediators was more commonly observed in critically ill patients, in addition to very low levels of lymphocytes in peripheral blood, especially natural Killer cells (NK), which demonstrate that the immunological status is closely related to the prognosis of the disease (Figure 2) (35–37).

In patients with COVID-19 with elevated inflammatory cytokines, the postmortem pathology revealed tissue necrosis, interstitial macrophages and monocyte infiltrations in the lung, heart and gastrointestinal mucosa. In addition, severe lymphopenia with hyperactivated proinflammatory T cells and decreased regulatory T cells are commonly seen in critically ill patients (10, 37, 38). Huang et al. measured the cytokine levels in 41 patients with COVID-19 and the results showed that significantly higher cytokine levels are observed in critically ill patients in different age groups in the presence or absence of comorbidities, as mentioned in other studies. Most critically ill patients with COVID-19 have a considerably high and persistent levels of erythrocyte sedimentation rate and immune mediators, being associated with acute respiratory failure syndrome, hypercoagulation and disseminated intravascular coagulation, manifested as thrombosis, thrombocytopenia and gangrene of extremities. It seems that the immune response worsens lung damage and leads to further complications (10, 36, 38–40).

These findings reveal that patients with COVID-19 are usually accompanied by increased immunological factors with inflammatory responses, justifying that the concentrations of immunological factors are associated with the severity of the disease. In fact, a storm of immune mediators is one of the clinical manifestations of COVID-19. These immune mediators act as pro-inflammatory agents, resulting in the cytokine release syndrome (CRS), being an important factor in the pathology of COVID-19 (39).

Corticosteroids were widely used during outbreaks of severe SARS-CoV1 and MERS-CoV2 and their use are now being considered in patients with COVID-19 in combination with other therapies (11). Corticosteroids have a good inhibitory effect on inflammatory factors and are often used as an auxiliary treatment for viral pneumonia, which is one of the reasons corticosteroids are being commonly prescribed for the treatment of patients with COVID-19 in the intensive care unit. Patients with COVID-19 are treated mainly with symptomatic therapy, however, corticosteroids are widely used in the symptomatic treatment of severe pneumonia (41, 42).

The main anti-inflammatory effect of glucocorticoids involves the inhibition of a high number of pro-inflammatory genes that encode cytokines, chemokines, cell adhesion molecules, inflammatory enzymes and receptors that ultimately address the inflammatory process (42). The use of glucocorticoids can improve early fever, promote the absorption of pneumonia and induce better oxygenation of the airways. However, some studies have shown no beneficial effect of glucocorticoids due to their adverse reactions and delay in eliminating the virus (43).

As described in the Chinese guidelines of COVID-19 (44), clinicians need to be cautious about steroid use due to its nebulous benefits in the scenario of viral respiratory infection. Several studies have reported inferior results in patients with SARS treated with corticosteroids (45) and, in the case of MERS-CoV coronavirus, the results showed that patients who received corticosteroids were more likely to need mechanical ventilation, vasopressors and renal replacement therapy (46). Another concern of corticosteroid use is their short- and long-term adverse effects that may lead to consequences such as joint pain and bone marrow abnormalities in patients with SARS (47).

Previous reports have shown that the use of corticosteroids can lead to prolonged removal of viral RNA from the airways (46), blood (48), and feces (49), resulting in longer hospitalization and ultimately increased mortality risk. The main concern is that corticosteroids may delay the elimination of the virus and increase the risk of secondary infection, especially in those with compromised immune system. In addition, biological agents targeting pro-inflammatory cytokines can only inhibit specific inflammatory factors and, therefore, may not be so effective for COVID-19 treatment in which other cytokines may be involved.

**Figure 3:**
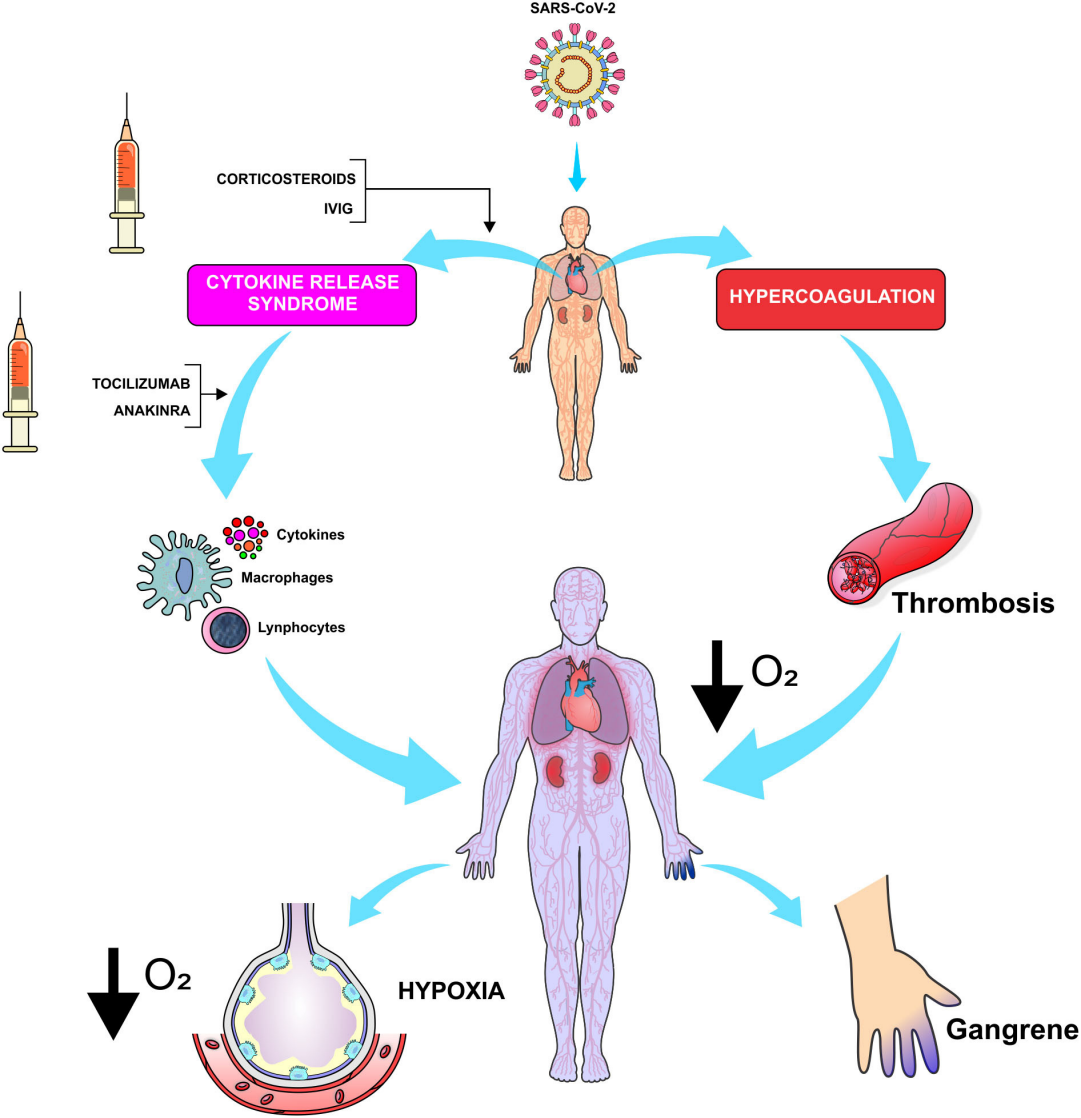
Considered as one of the most damaging effects of the pathophysiology of SARS-CoV-2, CRS determines a reduction in gas exchange in pulmonary alveoli while causing an increase in thromboembolic events that disrupt the lung tissue and reduce the respiratory reserve. The anti-inflammatory drugs reduce the levels of cytokines and improve the gas exchange between the alveolus and pulmonary capillary. In addition, they reduce the production of thrombin by the capillary endothelium. (O_2_, oxygen; IVIG, Intravenous immunoglobulin). *Figure source: Authors' own drawing*.

Siddiqi and Mehra (50) suggested that the target therapy in stage III of COVID-19 requires the use of immunomodulatory agents to reduce systemic inflammation. At this stage, the use of corticosteroids may be justified if combined with cytokine inhibitors, such as tocilizumab (IL-6 inhibitor) or anakinra (IL-1 receptor antagonist). Intravenous immunoglobulin (IVIG) can also play a role in modulating immune systems under hyper-inflammatory state (Figure 3). In general, the prognosis and recovery from this critical stage of the disease are poor and the rapid introduction of this therapy might result in some benefits (36, 50).

Wang et al. reported that 44.9% of patients with COVID-19 have received glucocorticoid therapy with no effective results (42). Russell et al. reported clinical evidences that did not support the treatment with corticosteroids in lung injury caused by COVID-19 (45). Due to the lack of enough evidences, the WHO provisional guidelines (February 22, 2020) do not support the use of systemic corticosteroids in the treatment of viral pneumonia and in suspected cases of COVID-19 (51). Therefore, the efficacy and adverse effects associated with the use of glucocorticoids in COVID-19 need to be elucidated.

A review of treatments for acute respiratory distress syndrome based on six studies with a total of 574 patients concluded that there is insufficient evidence to recommend treatment with corticosteroids. Observational data suggest increased mortality and higher secondary infection rates in influenza, as well as impaired clearance of SARS-CoV and MERS-CoV. Patients who received corticosteroid therapy were more likely to develop bacterial infection due to immunosuppression. This can worsen the disease and might lead to death (52, 53).

A team of frontline clinicians in China recommended administration of corticosteroids in low to moderate doses for a short period of time in critically ill patients with COVID-19 pneumonia (54). However, current WHO provisional guidelines (released on January 28, 2020) on the clinical treatment of severe acute respiratory infection in suspected cases of new coronavirus (SARS-CoV-2) advises against the use of corticosteroids, unless otherwise strictly indicated (51).

Other anti-inflammatory drugs, such as baricitinib, also block the production of IFN-γ, which is necessary for fighting the virus, and theoretically may not be suitable for the treatment of the inflammatory response caused by COVID-19. The time frame of anti-inflammatory treatment is very important and according to reports, critically ill patients generally experience abrupt deterioration within 1–2 weeks, which means that the immediate start of anti-inflammatory therapy in this extremely short time window is likely to achieve a favorable response (10, 52).

**Figure 4:**
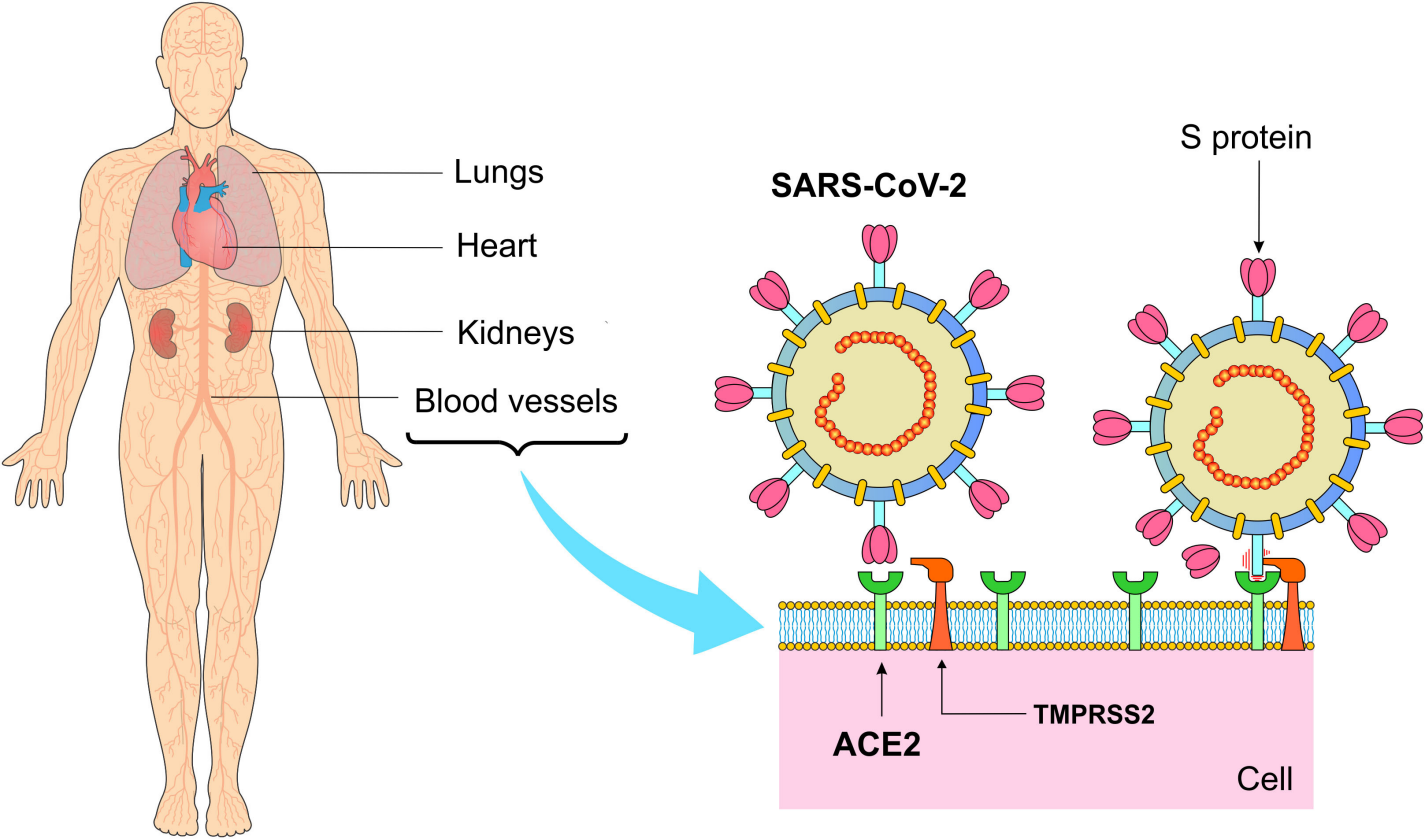
Main sites of angiotensin-converting enzyme 2 (ACE2) expression and binding of S protein to the ACE2 receptor after activation of the transmembrane serine protease 2 (TMPRESS2). ACE2 is a surface enzyme that acts as a port of entry of SARS-CoV-2 into the cells. This enzyme is present mainly in the pulmonary, cardiac, renal and vascular tissues, even though its presence in the adipose tissue places obesity as one of the risk factors for COVID-19. *Figure source: Authors' own drawing*.

The receptors for SARS-CoV-2 may be ACE2, which is a cell surface protein that exists widely in cells of the heart, kidney, blood vessels and especially in alveolar epithelial cells. SARS-CoV-2 can invade and enter these cells through endocytosis (Figure 4). One of the regulators of endocytosis is protein kinase 1 associated with AP2 (AAK1). AAK1 inhibitors can stop the virus from passing into cells and can be useful in preventing virus infections. Baricitinib, a JAK and AAK1 inhibitor, has been suggested as a possible candidate for the treatment of COVID-19, considering its relative safety and high affinity for ACE2. Therapeutic doses in the range of 2–4 mg once a day is enough to reach the plasma inhibitory concentration (52). However, as mentioned above, the biggest concern with JAK inhibitors is that it can inhibit a variety of inflammatory cytokines, including interferon, which plays an important role in controlling virus activity.

Tocilizumab (TCZ) is a recombinant human IL-6 monoclonal antibody, which specifically binds to membrane-bound IL-6 receptors (IL-6R), thereby blocking IL-6 signaling and its mediated inflammatory response. Along with basic antivirus treatment, TCZ was administered to 20 patients (400 mg once a day, intravenously) and within a few days, the fever returned to normal and other symptoms improved markedly. Seventy percentage showed improvement in oxygenation and the opacity lung injury on CT scans absorbed in 90.5% of the patients. In addition, the percentage of peripheral lymphocytes returned to normal in 52.6% of the patients. Their data suggest that TCZ may be an effective alternative for the treatment of critically ill patients with COVID-19 (9, 53, 54).

Previous reports have shown that the administration of corticoid therapy to patients with immunological disorders has improved their health status (55). The use of 6 mg/day of dexamethasone reduced the mortality rate of patients when compared to those without corticoid treatment. In addition, a reduction in mortality was observed in one third of the patients who received invasive mechanical ventilation and in one fifth who received oxygen without invasive mechanical ventilation. However, the treatment did not reduce mortality in those who did not receive respiratory support. These findings reveal that the use of dexamethasone (6 mg/day) for up to 10 days reduced mortality by 28 days in patients with COVID-19 who received invasive mechanical ventilation (55).

A randomized clinical trial compared the mortality rate between a group of patients treated with dexamethasone (2,104 patients) and another group treated with the usual care (4,321 patients). Overall, 482 patients (22.9%) in the dexamethasone group and 1,110 (25.7%) in the usual care group died 28 days after randomization. The proportional and absolute differences between groups in respect to mortality rate varied considerably according to the level of respiratory support at the time of randomization. In the dexamethasone group, the incidence of death was lower than that of the usual care group among patients with invasive mechanical ventilation (29.3 vs. 41.4%) and among those receiving oxygen without invasive mechanical ventilation (23.3 vs. 26.2%), but not among those who did not receive respiratory support at randomization (17.8 vs. 14.0%) (55).

Corticosteroid administration for a short period of time in patients with COVID-19 has improved the prognosis of the disease, resulting in decreased mortality and intubation (56). Such findings were corroborated by Selvaraj et al. (57) who reported that the short-term use of systemic corticosteroids by hospitalized patients with SARS-CoV-2 with hypoxic respiratory failure was well-tolerated and that the majority of the patients had an improvement in their prognosis. In addition, other studies have shown that short-term use of corticosteroids alleviates the severity of inflammation and reduces the mortality rate (38, 58). These findings support the use of corticosteroids during the ideal window of time to help alleviate the severity of inflammation and ultimately prevent the phase of severe hyperinflammation. However, a thorough clinical evaluation of each patient is mandatory before initiating corticosteroid therapy. Glucocorticoid treatment in patients with initial C-reactive protein (CRP) above 20 mg/dL has been associated with a significantly reduced risk of mortality and a decreased need for mechanical ventilation, whereas glucocorticoid treatment in patients with CRP below 10 mg/dL has been associated with significantly increased risk of mortality and higher need for mechanical ventilation (59).

There are several reports on the administration of anti-inflammatory drugs for the treatment of COVID-19. Some studies demonstrate the benefits of using corticosteroids in low doses for a short period of time during stage III of COVID-19. The use of corticosteroids needs to be well-evaluated, as other drugs can treat hyperthermia and inflammatory processes more selectively. In general, many studies indicate that there is no single reason to expect patients with SARS-CoV-2 infection to benefit from corticosteroids use but are otherwise more likely to be harmed by this treatment. Based on the studies analyzed in this review, corticosteroid administration to patients with COVID-19 may more likely inhibit immune responses and increase the rate of bacterial infection, which would probably prolong hospitalization and increase mortality rate.

### Antivirals and Their Use in the Context of SARS-CoV-2

In order to find specific antiviral treatment for the new coronavirus, tests with broad spectrum drugs have been carried out. In addition, a screening of existing chemicals capable of affecting the transcription mechanisms from different cell perspectives has identified some promising drug candidates. Finally, the development of new specific antiretroviral drugs based on the genomic characteristics and viral behavior of SARS-CoV-2 has also been considered (60).

The use of interferon-alpha (IFN-α) at 5.000 U twice a day (bis in die—BID) and lopinavir/ritonavir (400/100 mg twice a day through oral route) has been recommended by Chinese guidelines. IFN-α is a broad-spectrum antiviral while lopinavir is a protease inhibitor and ritonavir enhance lopinavir activity by increasing its half-life. The use of lopinavir/ritonavir showed to be more effective than ribavirin alone as the patients treated with the former association showed lower rates of progression to acute respiratory distress syndrome (ARDS) and mortality (20, 61).

#### Nucleoside Analogs

Favipiravir and ribavirin are the main nucleoside analogs, whose mechanism of antiviral activity involves the induction of lethal mutagenesis of some viruses, such as influenza. In fact, the use of favipiravir associated with oseltamivir in the treatment of severe influenza showed to be more effective than oseltamivir alone (21). However, there is an exonuclease expression in non-structural protein 14 (nsp14-ExoN) in the coronavirus families, which may point to SARS-CoV-2 being resistant to this category of antivirals, corroborating studies in which ribavirin and favipiravir showed relatively poor activity against coronavirus (62).

**Figure 5:**
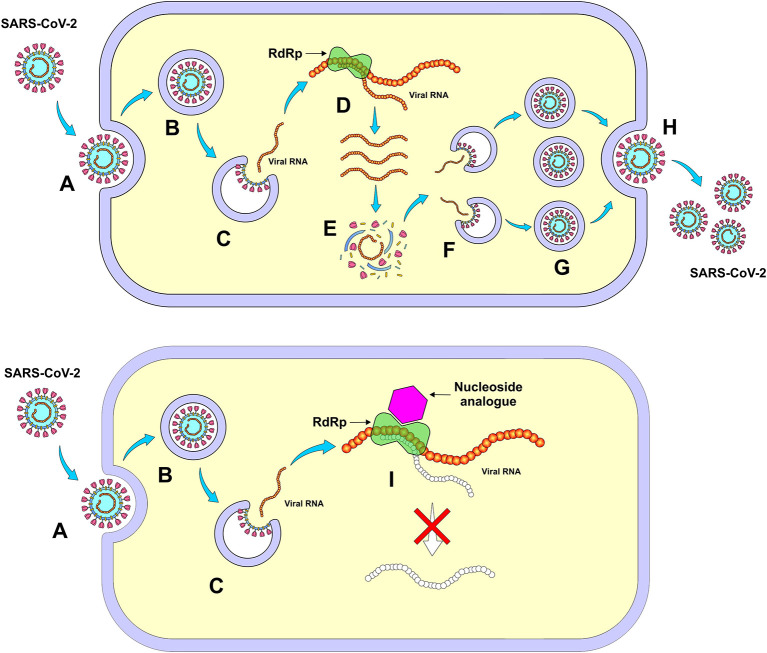
Mechanism of action of nucleoside analogs (Remdesivir) through inhibition of RNA-dependent RNA polymerase (RdRp). **(A)** Entry of SARS-CoV-2 into the cell, **(B)** Formation of the endosome, **(C)** Release of viral RNA in the cell's cytoplasm, **(D)** Viral self-replication by RdRp, **(E)** Synthesis of virus structural proteins, **(F)** Incorporation of RNA viral, **(G)** New viral units, **(H)** Release of new viruses, and **(I)** Inhibition of viral self-replication by RdRp. *Figure source: Authors' own drawing*.

Remdesivir is another nucleoside analog that has shown previous activity against Ebola and Nipah virus infection, whose mechanism of action involves the inhibition of RNA-dependent RNA polymerase (RdRp), therefore, it can inhibit the replication of coronaviruses. In fact, remdesivir has demonstrated activity against both SARS and MERS, whose antiviral effect might potentially be extrapolated to SARS-CoV-2 (Figure 5) (63).

A molecular docking study was carried out involving ribavirin, remdesivir, sofosbuvir, galidesivir and tenofovir against SARS-CoV-2 RdRp. These are anti-polymerase drugs that are currently on the market and have been previously approved for use as antivirus. All five drugs were able to tightly bind to the new coronavirus strain RdRp and therefore, are considered promising candidates to treat COVID-19 (64).

Remdesivir has shown a promising antiviral effect against COVID-19 in mild to moderate clinical situations. In an experimental animal study, the rodent groups infected with the Middle East Respiratory Syndrome (MERS)-CoV that received remdesivir showed an effective reduction in viral load when compared to the control group. An improvement in the damage to the lung parenchyma was observed, which promoted better local tissue recovery compared to the group treated with lopinavir and ritonavir in combination with IFN-β (64).

In a randomized phase 3 study, whose inclusion criteria included SARS-CoV-2 infection and O_2_ saturation equal to or <94% with pneumonia, 200 mg of remdesivir was administered intravenously on day 1 and 100 mg on subsequent 5 or 10 days. Three hundred ninety-seven patients were used in this study, 200 under a 5-days regimen and 197 under a 10-days regimen. In general, patients who underwent 10-days treatment had a significant clinical worsening in relation to the 5-days group. Most common adverse reactions were nausea (9% of reports), worsening of respiratory failure (8%), elevation of alanine aminotransferase level (7%), as well as constipation (7%) (22).

Sixty four percentage of the patients from the 5-days regimen group had recovered, compared to 54% from the 10-days group. However, this study has some limitations such as the absence of a randomized and placebo-controlled trial as well as the lack of analysis of viral load for SARS-CoV-2 during and after treatment. In addition, this study showed no significant difference in terms of efficacy between 5 and 10 days of treatment with intravenous remdesivir in patients with severe COVID-19 who did not need mechanical ventilation. For patients with the need for mechanical ventilation, the 10-day regimen was more effective, although it needs more in-depth studies among risk groups and immunocompromised patients in order to identify the effectiveness of the shorter-duration treatment (22).

An investigator-initiated, randomized, placebo-controlled, multicentered, double-blinded trial was conducted using intravenous remdesivir in 155 patients that were positive for SARS-CoV-2 and had chest imaging suggestive for pneumonia, oxygen saturation of 94% and FIO_2_ ≤ 300 mmHg. Patients received intravenous remdesivir (200 mg/day on the first day and 100 mg/day on days 2–10), whereas the placebo group received the same infusion volume of a placebo solution for a total of 10 days. The results showed that the rate of clearance of the virus and mortality were not significantly changed in the group treated with intravenous remdesivir, however, an overall reduction of nearly 5 days in the median time of improvement of the clinical manifestations was observed. There was no significant reduction in the viral loads and in the duration of invasive mechanical ventilation. There were reports of adverse events in 66% of the patients in the remdesivir group and in 50% of those in the placebo group (23). It is important to emphasize the need for additional studies with larger samples, as well as strategies to enhance the effectiveness of remdesivir by either using higher doses or associating with other antivirals/antibodies that could possibly neutralize SARS-CoV-2.

#### Neuraminidase Inhibitors

Chen et al. (24) studied the use of neuraminidase inhibitors such as oseltamivir (75mg twice a day through oral route) in 75 patients receiving treatment with non-specific antivirals such as ganciclovir (guanine nucleotide analog, 0.25 g twice a day intravenously) and the aforementioned association of lopinavir with ritonavir (500 mg twice a day, oral). Associations with antibiotics of different classes, as well as with corticosteroids were also investigated but with no description about the specific outcome in relation to the hospitalization period and death rate (24).

The use of neuraminidase inhibitors such as oseltamivir and zanamivir can influence influenza-like manifestations resulting in a decrease in the duration of symptoms. They can be used in situations of mild respiratory manifestations and therefore, it might be considered as a preventive measure among the strategies for flattening the curve and for preventing the collapse of health systems due to non-specific problems related to COVID-19 (65). On the other hand, since the new coronavirus does not synthesize neuraminidase (62), it seems likely to infer that this class of antivirus might not be effective for SARS-CoV-2.

#### Ivermectin

**Figure 6:**
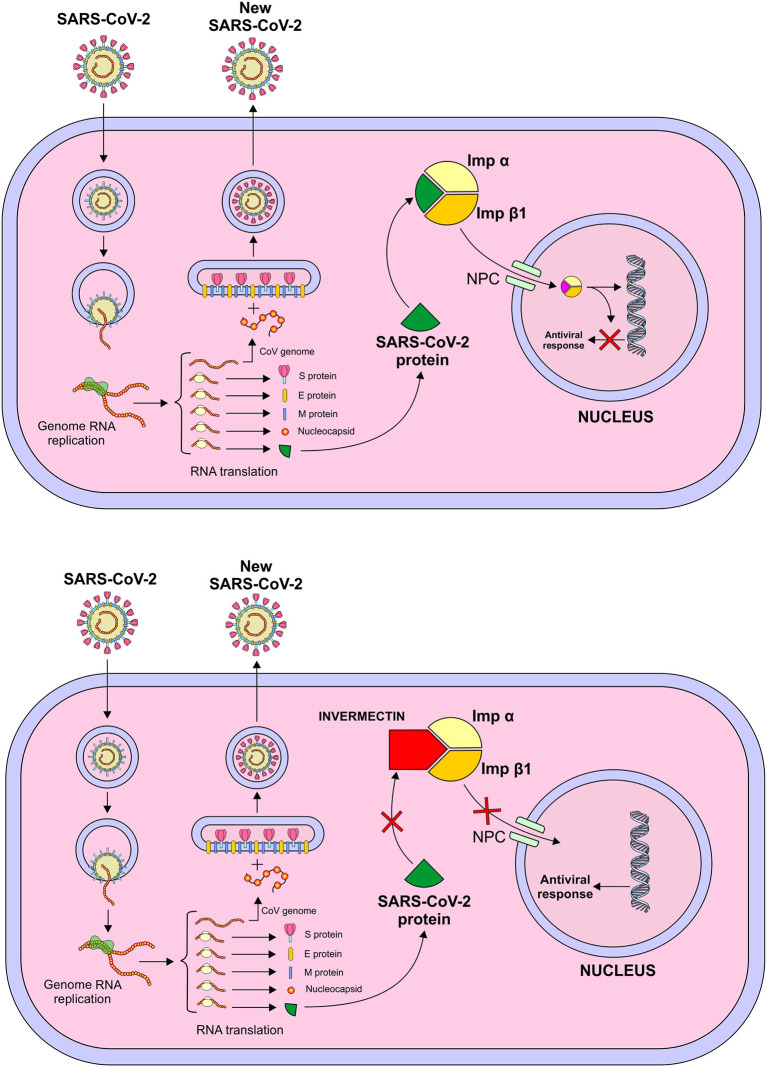
Mechanism of action of Ivermectin – Inhibition of integrase protein and importin α/β1 (IMPα/β1) heterodimer which helps the former to be inserted into the nucleus during the interaction between the virus and the human cell, resulting in interruption of viral replication. *Figure source: Authors' own drawing*.

Ivermectin's antiviral activity has been proven *in vitro* against several viruses, including influenza, dengue, viral encephalitis and HIV. It acts by inhibiting the integrase protein and importin α/β1 (IMPα/β1) heterodimer which helps the former to be inserted into the nucleus during the interaction between HIV-1 and the human cell, resulting in interruption of viral replication (Figure 6). It is believed that the activity of IMPα/β1 on RNA viruses is what designates the broad spectrum of ivermectin (66). In addition, ivermectin stimulates GABA-gated chloride channels that ends up triggering a hyperpolarization process, resulting in paralysis of the infecting organism. Another proposed mechanism involves the immunomodulation of the host's response through the activation of neutrophils, with increased levels of C-reactive protein and IL-6 (67).

Another study investigated the hypothesis that IMPα/β1 influences the closure of the signal-dependent nucleocapsid protein of the SARS-CoV-2 nucleus-cytoplasmic, which impacts on the division of the host cell. Accessory protein ORF6 plays a role in antagonizing the activity of the transcription factor STAT1 by capturing IMPα/β1 in the membrane of the rough endoplasmic reticulum/Golgi membrane. This study was undertaken by infecting Vero/hSLAM cells with SARS-CoV-2 from the isolated strain Australia/VIC01/2020, followed by the addition of 5 μM of ivermectin. On days 0–3, supernatant cell materials were collected for RT-PCR analysis for SARS-CoV-2. As a result, a 93% reduction in the viral supernatant RNA (indicative of released virions) was found after 24 h. At 48 h, the reduction in viral RNA in the ivermectin-treated group increased to approximately 5,000 times compared to the control group, with no viral replication within 72 h (25).

Despite the broad antiviral spectrum of ivermectin found mostly *in vitro*, it is necessary to emphasize that clinical trials need to be conducted in order to better correlate the results of animal models to humans. It is worth noting that there was no evidence of reproducibility of the results found in infected rat models, which reinforces the FDA statement in April 2020 about the risks of self-medication with ivermectin against COVID-19. *In vitro* studies with promising results represent only the first stage of drug development. In addition, the studies that showed the efficacy of ivermectin on SARS-CoV-2 used doses within the microgram range. On the other hand, in humans, serum ivermectin levels for a safe therapeutic window should be around 20–80 ng/mL, which are considerably lower than those used in the *in vitro* experiments (68).

#### Nitazoxanide

Nitazoxanide has been considered as a therapeutic option for SARS-CoV-2. It is an antiparasitic agent approved by FDA for the treatment of Cryptosporidium and Giardia, in addition, it has shown a broad-spectrum antiviral activity against Noro and Rotavirus, as well as hepatitis B and C. Its mechanism of action is based on the increase in the sensitivity to cytoplasmic RNA and Interferon I pathways, which implies in regulating specific host cellular mechanisms dodged by the virus for its replication (69).

An ongoing clinical trial has shown the antiviral activity of nitazoxanide against 16 viruses including Influenza A subtypes (H1N1, H3N2, H3N2v, h3n8, h5n9, h7n1), Influenza B, respiratory syncytial viruses, dengue fever, yellow fever, Japanese encephalitis virus, rotavirus, HIV, SARS and MERS. A randomized, double blind study with 86 participants is currently being undertaken in Mexico where it compares the use of Hydroxychloroquine (200 mg, 12/12 h for 10 days) vs. Hydroxychloroquine + Nitazoxanide (200 + 500 mg, 12/12 h for 10 days). This latter association aims to decrease the hyperinflammatory process that leads to the evolution of the respiratory condition (26).

### Azithromycin

**Figure 7:**
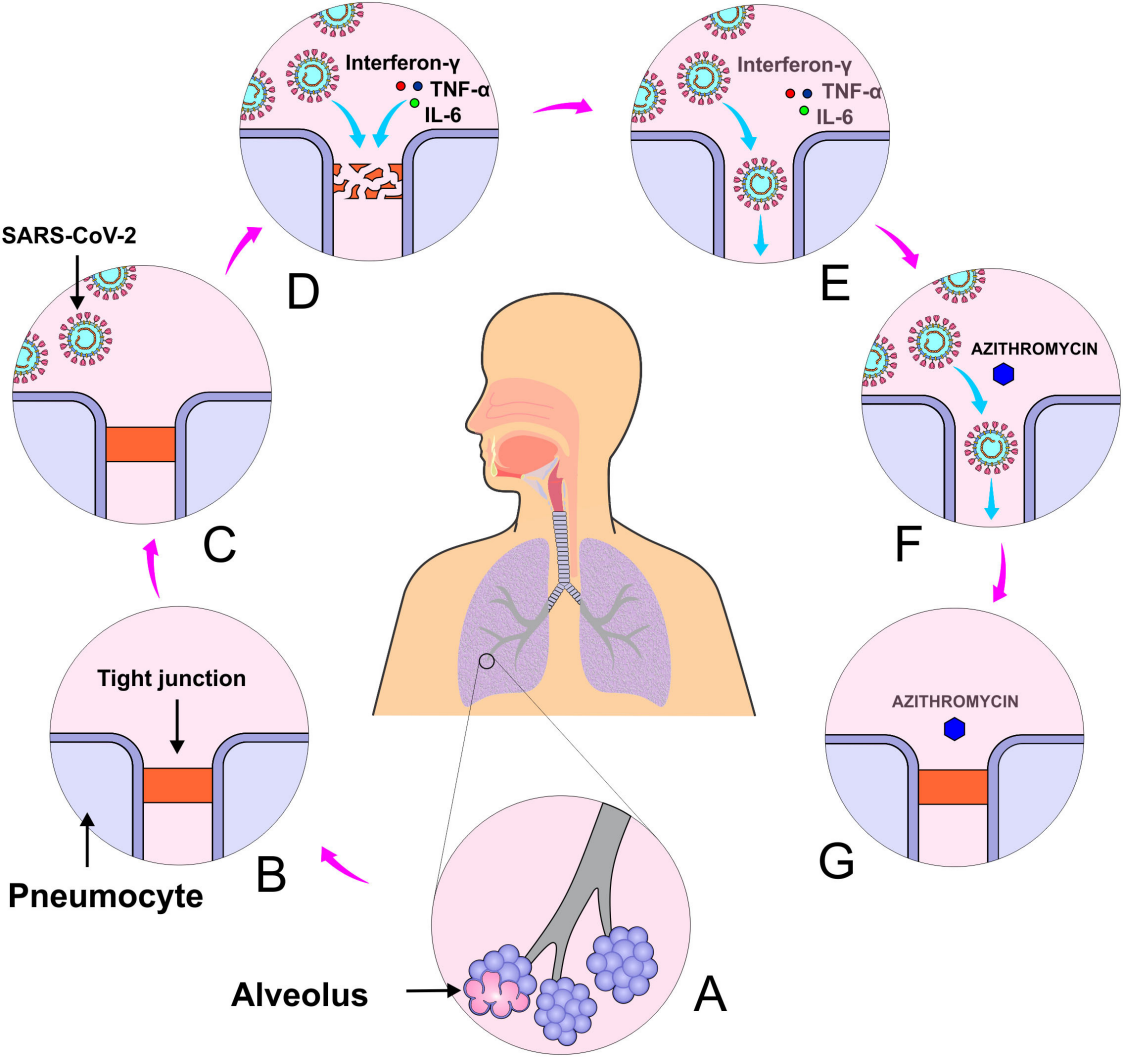
Azithromycin activity in maintaining the integrity of tight junctions. **(A)** Alveoli, **(B)** Tight junction, **(C)** Lung infection by SARS-CoV-2, **(D)** Tight junction disruption caused by virus interleukin 6, tumor necrosis factor and interferon α, **(E)** SARS-CoV-2 entrance, **(F)** Administration of azithromycin, and **(G)** Restoration of tight junctions.

Epithelial tight junction functions as a barrier that impedes the entrance of pathogenic microorganisms to several organs and tissues, such as lungs. Coronaviruses seem to disrupt the epithelial tight junctions by downregulating proteins involved in the maintenance of their integrity, which increases the potential for SARS-CoV-2 invasion and penetration. Conditions known to increase risk for COVID-19 complications include advanced age, diabetes mellitus, smoking and chronic lung disease. All these conditions are associated with higher predisposition to dysfunction of tight junctions. Moreover, SARS-CoV-2 infected patients have been reported to have increased levels of IL-6, TNF-alpha and interferon-gamma, all of which have shown to impair tight junctional function in several epithelial cell lines (Figure 7) (70).

It is believed that azithromycin can limit the growth of pathogens that disrupt intercellular tight junctions. This drug alters the processing and location of natural sealant molecules, which seems to exhibit a sealing effect on respiratory tight junctions (71). In addition, azithromycin has long been used to prevent respiratory tract infections caused by virus (72). It has shown to be effective against some viruses such as influenza (73), Zika (74), and Ebola (75).

Some preliminary data is available to support the use of azithromycin with hydroxychloroquine for treatment of patients with COVID-19, even though the success of its use may be limited to patients at the peak of COVID-19 symptoms and in potential respiratory collapse. A clinical study conducted in France showed that the association of hydroxychloroquine (600 mg/day for 10 days) with azithromycin (500 mg/day on the first day followed by 250 mg/day on the next 4 days) was advantageous as 100% of the individuals treated with this association were virologically cured compared with 57.1% of those treated with only hydroxychloroquine (12). Despite its small sample size (26 patients in the treated group and 16 in the control group), this study opens the possibility of a synergistic effect of the combination of azithromycin with hydroxychloroquine.

Gabriels et al. (76) advert that the combination of hydroxychloroquine and azithromycin can prolong the QT interval and therefore may increase the arrhythmogenic risk of the patients submitted to such treatment. The authors raised the importance of cardiac rhythm monitoring in SARS-CoV-2 positive patients under hydroxychloroquine + azithromycin treatment, especially those with prior history of atrial fibrillation.

Although azithromycin has shown potential activity in maintaining the integrity of pulmonary epithelial tight junctions, a question arises whether it could aid in ameliorating pulmonary compromise in those patients in which SARS-CoV-2 has already penetrated the respiratory epithelium with the patient exhibiting pulmonary complications. Therefore, it seems likely to infer that the sealing activity of azithromycin on respiratory epithelial tight junctions might be useful for prophylaxis of COVID-19. In addition, its widespread use during this pandemic wave might increase the risk of antibiotic resistance and therefore, the pay-off must be worthwhile.

The use of azythromycin in combination with hydroxychloroquine has been investigated. Retrospective study involving 2,541 hospitalized patients with a mean age of 64 revealed a significant reduction in mortality among those who received the combination of hydroxychloroquine and azithromycin. However, the authors emphasize that prospective studies are essential to confirm the impact of such association in comparison with the use of hydroxychloroquine alone (77).

### Chloroquine and Hydroxychloroquine

**Figure 8:**
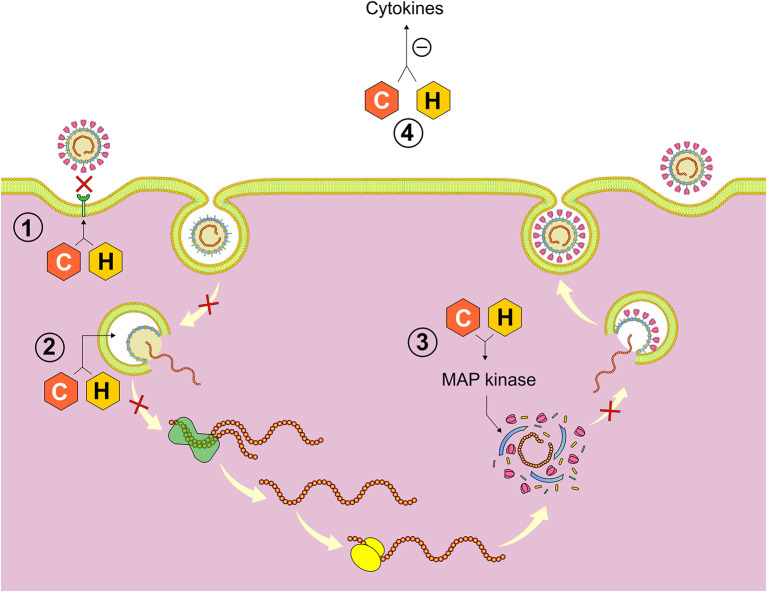
Mechanisms of action of chloroquine (C) and hydroxychloroquine (H). (1) Inhibition of host glycosylation receptor and quinone reductase 2 responsible for the formation of the sialic acid necessary for the incorporation of the virus into the host cell, (2) Alteration of the endosome pH and inhibition of cathepsins responsible for the extrusion of the viral RNA of the endosome, (3) MAP kinase inhibition interfering with the proteolytic processing of protein M, and (4) Immunomodulatory effect resulting in inhibition of the synthesis of cytokine. *Figure source: Authors' own drawing*.

Chloroquine and hydroxychloroquine are drugs derived from 4-aminoquinolines that have been reported to inhibit SARS-CoV2 by blocking viral entry through the inhibition of host receptor glycosylation, as well as through proteolytic processing and endosomal acidification. In addition, immunomodulatory effects have been attributed to these drugs through inhibition of cytokine production, autophagy and lysosomal activity in host cells (Figure 8). Hydroxychloroquine seems to have a greater antiviral activity (EC50 = 0.72 μM) besides having more tolerable safety profile, which makes it the preferred drug to treat malaria and autoimmune diseases such as rheumatoid arthritis, lupus erythematosus and dermatological conditions caused or aggravated by sunlight (78).

Based on preliminary results (79) carried out since January 2020, Chinese, South Korean, American and Brazilian health authorities have recommended the use of chloroquine and hydroxychloroquine for the treatment of SARS-CoV-2. Some clinicians consider their use reasonable in hospitalized patients with severe illness due to SARS-CoV-2 who are not eligible for hydroxychloroquine and chloroquine trials (80, 81).

In a randomized double-masked, phase IIb clinical trial (ChlorCovid-19 Study), with 81 adults who were hospitalized with SARS-CoV-2, preliminary findings suggest that the highest dose of chloroquine (600 mg, 2x/day, for 10 days, total dose of 12 g) should not be recommended for critically ill patients with COVID-19 due to security risk. These findings prematurely interrupted the recruitment of patients for this study (17, 82, 83).

The ideal dosage is uncertain; the FDA suggests hydroxychloroquine 800 mg on the first day followed by 400 mg daily and chloroquine 1 g on day 1 followed by 500 mg daily. Duration of treatment varies from four to seven days depending on the clinical response. Other hydroxychloroquine regimens includes: 1–400 mg twice a day on day 1 followed by 400 mg once a day for 5 days; 2–400 mg twice a day on day 1 and 200 mg twice for 4 days and 3–600 mg twice daily on day 1 and 400 mg daily for four days (84, 85).

The clinical data available for the use of hydroxychloroquine and chloroquine against COVID-19 is limited and its effectiveness is so far unknown. In the United States, the FDA has authorized the emergency use of these drugs in adolescents and adults hospitalized by COVID-19 when participation in clinical trials is not feasible. However, if these agents are used outside a clinical trial, the possibility of drug toxicity (including prolongation of the QTc interval, drug-induced *torsades de pointes*—a form of polymorphic ventricular tachycardia, as well as cardiomyopathy and retinal toxicity) are more likely to occur. Gastrointestinal responses, such as vomiting and diarrhea, are the most common adverse effects of these two drugs. Previous reports have shown that patients with long-term exposure to chloroquine suffer from severe side effects, such as retinopathy, circular defects (or bull eye maculopathy) and diametric defects in the retina. In addition, drug interactions should be considered before use, especially in individuals who may be at risk. Thus, more susceptible patients should be monitored closely for side effects during chloroquine/hydroxychloroquine use. In fact, the American College of Cardiology suggests QTc monitoring in those patients at risk under the use of these drugs (80, 86–89).

Recent retrospective studies show a reduction in mortality among COVID-19 hospitalized patients who received the combination of hydroxychloroquine and azithromycin compared to those who underwent other treatments (13). However, a recently published multicenter randomized controlled trial with 504 patients with mild-moderate COVID-19 showed that the use of hydroxychloroquine at a dose of 400 mg twice daily alone or with azithromycin at a dose of 500 mg once daily for 7 days did not improve clinical status at 15 days when compared with standard care (16).

Two recently published randomized controlled trials investigated the use of hydroxychloroquine in reducing the severity of symptoms in adults with mild COVID-19. In the first study (15), 491 adults, of which 341 had laboratory-confirmed infection, were randomized into 2 groups that received either 800 mg of hydroxychloroquine on the first day and 600 mg/day on the next five days or equivalent doses of placebo. In the second study, 136 adults were randomly treated with 800 mg of hydroxychloroquine on the first day and 400 mg/day on the following six days and 157 did not receive this treatment (14). Both studies showed that hydroxychloroquine was not effective in reducing the severity of symptoms and that the rate of hospitalization was not significantly lower among those not treated with this drug.

Based on these findings, randomized clinical trials have not proven the efficacy of hydroxychloroquine alone or combined with azithromycin in reducing the duration and severity of symptoms in adults with mild-moderate COVID-19.

### Convalescent Plasma

Plasma therapy consists in administering to patients with infectious diseases and severe conditions the plasma itself, or fractionated antibodies, along with other immunoglobulins obtained from donors who are in the stage of convalescence of the infection or have been cured. This therapy has been used since the Spanish flu pandemic between 1917 and 1918, as well as in other pandemics due to infectious diseases, being the last record of studies in the Ebola epidemic between 2013 and 2015 (5, 90).

A study carried out with 69 Ebola infected patients (44 receiving plasma therapy) between 2014 and 2015 revealed a significant reduction in viral load after 24 h of treatment, even though no significant reduction in mortality was observed. However, the authors highlighted the promising effects of this therapy, although the study sample was small and randomized clinical trials with a larger sample is needed in order to confirm its efficacy (91).

During the first epidemic of SARS caused by coronavirus between 2002 and 2003, several studies involving plasma therapy were published, and the majority revealed a significant reduction in viral load and improvement of symptoms among treated patients (5). Subsequently, a meta-analysis involving 32 studies with patients infected with influenza and SARS coronavirus showed that convalescent plasma therapy was safe and may have reduced the mortality of these patients even though the study was biased and the quality of the evidence was low. In fact, the authors recommended conducting clinical trials with an appropriate methodology to better assess the effectiveness of this therapy (92).

To date, two studies have been published on the use of convalescent plasma in patients infected with the new coronavirus. Shen et al. evaluated a series of 5 cases of SARS-CoV-2 with severe symptoms characterized by pneumonia with rapid progression, PaO2/FiO2 ≤ 300 mmHg, under mechanical ventilation and with high viral load despite treatment with antivirals. All patients were treated with transfusions of convalescent plasma and it was demonstrated that body temperature was normalized in 4 patients after 3 days, whereas a decrease in the Sequential Organ Failure Assessment (SOFA) score, negative viral load and increased PaO2/FiO2 were observed after 12 days of treatment. However, the authors recommended more robust clinical trials in order to confirm these findings (93).

In the second study, 10 critically ill patients infected with the new coronavirus received a transfusion of 200 mL of convalescent plasma donated by people who had recently recovered from infection with antibody titers above 1:640. The authors stated that there was a significant improvement in oxygen saturation after 3 days, a decrease in C-reactive protein, varied absorption of lung lesions in radiological exams after 7 days and undetectable viral load in 7 patients without any serious side effect. However, they highlighted the need for randomized clinical trials with the purpose of defining the ideal dose and the best time for administration of convalescent plasma (19).

Finally, Roback and Garner stated in a recently published editorial that the use of convalescent plasma is not new as it had been tested in the pandemics of avian influenza (H5N1), influenza in 2009 (H1N1) and Ebola. The study by Cheng et al., who in 2003 tested the therapy in Honk Kong patients with SARS coronavirus, found that among the 80 patients who received plasma transfusions, the mortality rate was significantly lower than the 299 who did not receive the treatment. However, considering its use in cases of SARS-CoV-2, the authors warned that the administration of convalescent plasma has not yet been evaluated in randomized clinical trials. Therefore, it cannot be guaranteed that the improvement in the symptoms was due only to this intervention as the patients received additional drugs, such as corticosteroids and antivirals, that may have influenced the improvement of the condition. On the other hand, they agreed that the study published by Shen et al. provides sufficient evidence for large clinical trials involving the administration of convalescent plasma to critically ill patients with COVID-19 (19, 44, 93, 94).

The use of convalescent plasma associated with anticoagulants has been considered for patients with severe or life-threatening COVID-19 symptoms. However, a systematic review with 8 studies (no randomized clinical trial) and with 32 patients concluded that due to the high risk of bias and the low quality of evidence there is no certainty of the effectiveness and safety of convalescent plasma for hospitalized patients (95).

A randomized clinical trial that investigated the use of convalescent plasma in comparison with a standard treatment in 103 critically ill hospitalized patients showed no significant difference in the meantime for clinical improvement considering 28 days of follow-up (96). In this study, plasma was collected from adult donors aged between 18 and 55 with two negative PCRs before hospital discharge, who were asymptomatic and had left the hospital for more than 2 weeks. The authors pointed out that as the trial was terminated early, this study may not have enough power to detect an important clinical difference.

Spyropoulos et al. based on retrospective studies with hospitalized patients receiving anticoagulants, recommended the use of these drugs especially in those with high D-dimer (97). Likewise, in an article authored by an international collaboration of clinicians and investigators, the use of anticoagulant therapy to severe or life-threatening COVID-19 patients is recommended (98). However, there is a consensus in all these studies that randomized clinical trials are necessary in order to prove the effectiveness of plasma and anticoagulants.

### Vitamins

In supportive care, it is recommended the continuous assessment of nutritional status of all patients infected with SARS-CoV-2 in which those at nutritional risk should receive nutritional support as soon as possible. It is also emphasized that even patients with COVID-19 who are not at risk of malnutrition should maintain an adequate intake of proteins (1.5 g/day) and calories (25–30 kcal/day). In addition, some vitamins and oligoelements may have the potential to benefit infected patients due to their anti-inflammatory, antioxidant and anti-viral properties (99).

Virtual screening and other computational techniques have been used to discover drugs against SARS-CoV, dengue and Ebola viruses. Kandeel and Nazawi (100) used virtual screening to access the binding ability of 20 FDA approved molecules including a broad-spectrum antiviral (ribavirin), anti-hepatitis B (telbivudine) and two vitamins (vitamin B12 and nicotinamide) to a crystal structure of SARS-CoV-2 main protease. The evaluated parameters included the docking scores, ligand efficiency as well as lipophilic and hydrogen bonding interactions. The results showed that vitamin B12 and nicotinamide were ranked at the 4th and 6th position, respectively. Although the authors suggest that both vitamins have potential to be used for COVID-19 treatment in combination with other drugs, *in silico* modeling needs to be validated *in vitro*.

Previous reports have shown that vitamin C is a promising alternative to reduce the susceptibility of high-risk individuals to infection of the lower respiratory tract under certain conditions (101). Therefore, a moderate amount of vitamin C supplementation may be a way to prevent COVID-19. In addition, it has been shown that reduced levels of vitamin D and vitamin E in cattle can lead to bovine coronavirus infection (102). This suggests that adequate supplementation of vitamin D and vitamin E must be tested in humans in order to verify whether they may increase human resistance to SARS-CoV-2.

One of the most deleterious consequences of the prolonged indoor stay (lockdown) during this COVID-19 pandemic is the reduced levels of circulating vitamin D as a result of the insufficient sunlight exposure. Low levels of vitamin D has been associated with higher susceptibility to infections. The receptors for vitamin D are highly expressed by several immune cells, such as monocytes as well as T and B lymphocytes. Therefore, vitamin D deficiency is associated with significantly higher risk of respiratory viral infection, which means that increased vitamin D intake must be considered as an additional prophylactic measure for SARS-CoV-2 respiratory infection. Adequate levels of vitamin D might be achieved by administering this vitamin as a dietary supplement or by consuming foods with relatively high content of vitamin D, such as fatty fish, cod liver oil and egg yolks (102).

The use of vitamin D is justified by the growing evidence that normal values of this vitamin in infected patients can enhance immunity against pathogen and improve immune recovery during treatment with antiretroviral (99, 102–107). In addition, previous studies have demonstrated the role of vitamin D in preventing asthma and in improving the severity of asthmatic symptoms (108). A systematic review and meta-analysis published in 2017 identified 25 eligible randomized, double-blind, placebo-controlled trials with a total of 11,321 participants, whose effect of vitamin D supplementation on the risk of acute respiratory tract infection was assessed. This study showed that vitamin D supplementation was able to significantly reduce the risk of acute respiratory tract infection in 100% of the participants, especially those with considerably low 25-hydroxyvitamin D levels. It is interesting to note that such protective effect was more pronounced in individuals who received daily doses of vitamin D instead of large boluses. This latter procedure has been associated with reduced efficacy of vitamin D and increased risk of adverse outcomes (109).

**Figure 9:**
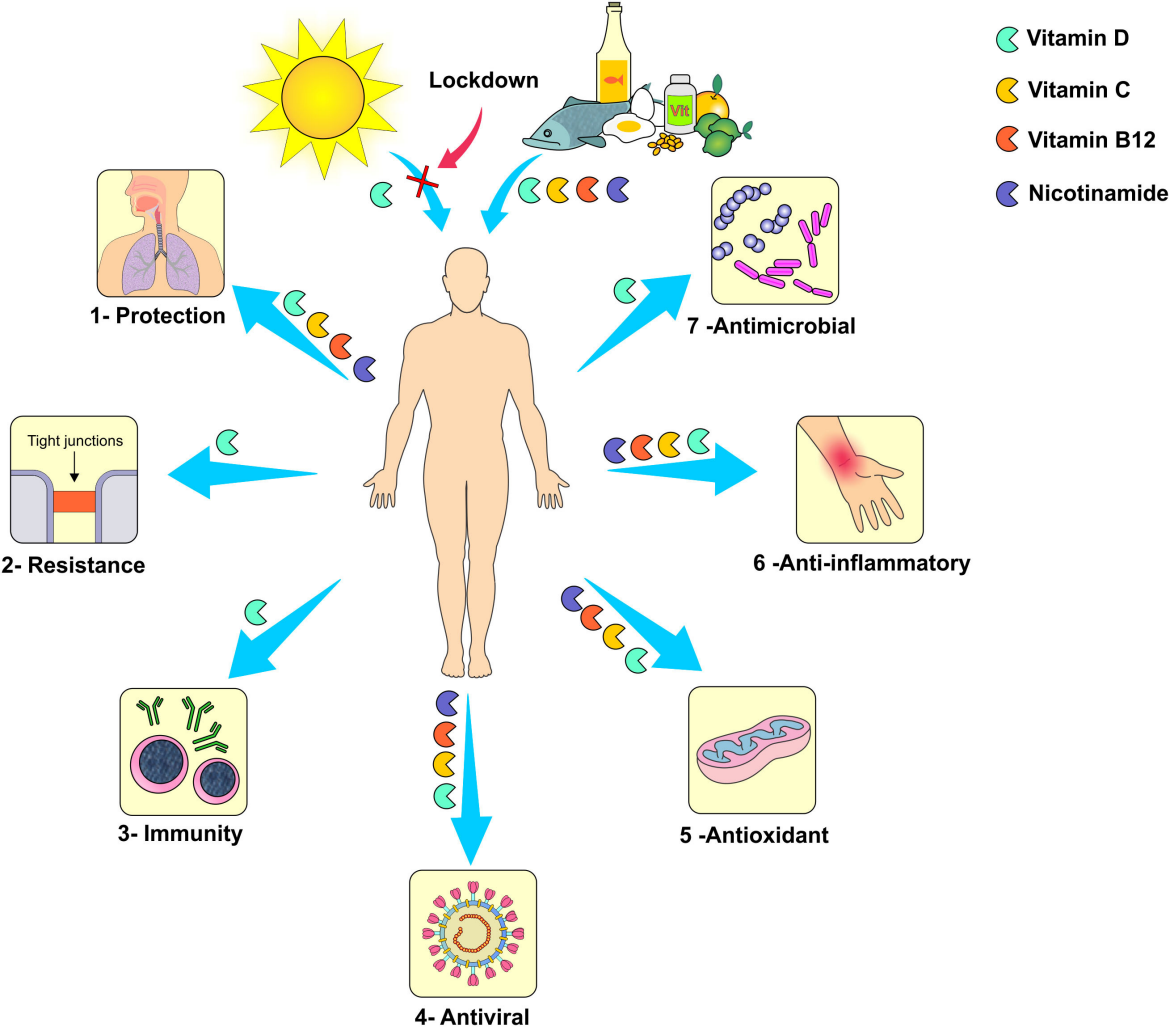
Mechanism of action of vitamins: 1- Reduction of susceptibility to viral infections of the respiratory tract, 2- Maintenance of the integrity of intercellular tight junctions resulting in increased resistance to microorganisms penetration, 3- Improvement of immunity, 4- Direct antiviral activity, 5- Antioxidant activity, 6- Anti-inflammatory activity, and 7- Increased production of antimicrobial peptides. *Figure source: Authors' own drawing*.

*In vitro* studies have shown that vitamin D actively participates in the respiratory homeostasis by increasing the expression of antimicrobial peptides and by affecting the replication of respiratory viruses. In addition, vitamin D preserves tight junctions, eradicates enveloped viruses by inducing cathelicidin and defensins and decreases the proinflammatory cytokines by the innate immune system, which prevents the cytokine storm that leads to pneumonia (Figure 9) (110). In fact, The British Medical Journal has recently published an editorial where many researchers included vitamin D deficiency as one of the putative risk factors for the novel coronavirus infection (111). Regarding the adequate dose of vitamin D, it depends on the severity of the hypovitaminosis D. It is recommended that the levels of circulating 25-hydroxyvitamin D should fall in the 40–60 ng/mL range for optimum protection against acute viral infections (112).

Patients with a deficit of 25-hydroxyvitamin D [25 (OH) D] should promptly have vitamin D supplied according to the results of blood tests [50,000 IU/week when levels of 25 (OH) D <20 ng mL; 25,000 IU/week when levels of 25 (OH) D are between 20 and 30 ng/mL] (13, 15, 16, 84, 89). On the other hand, a recent review study stated that for people at risk for influenza and/or COVID-19 a daily dose of 10,000 IU of vitamin D for a few weeks should be considered and once the levels of 25(OH)D increases, the daily dose of vitamin D should decrease to 5,000 IU (27).

Although several studies have demonstrated the role of vitamin D in the maintenance of immune homeostasis, a randomized controlled trial is still needed in order to confirm that adequate vitamin D intake can prevent respiratory tract viral infections such as that caused by SARS-CoV-2. In addition, it is worth to point out that vitamin D supplementation should take place under proper medical supervision as hypervitaminosis D can result in irreversible calcification of soft tissues and although rare, it can be life-threatening.

### Zinc

Zinc is an essential trace element that plays an important role in direct antiviral and immune responses. Such evidence can be confirmed by the higher risk of viral infections (Herpesviridae, HIV and Hepatitis C) in individuals with zinc deficiency. *In vitro* demonstration of the multiple mechanisms of antiviral actions of zinc has led to the indication of its supplementation as a preventive or therapeutic strategy to control viral infections (113–115).

**Figure 10:**
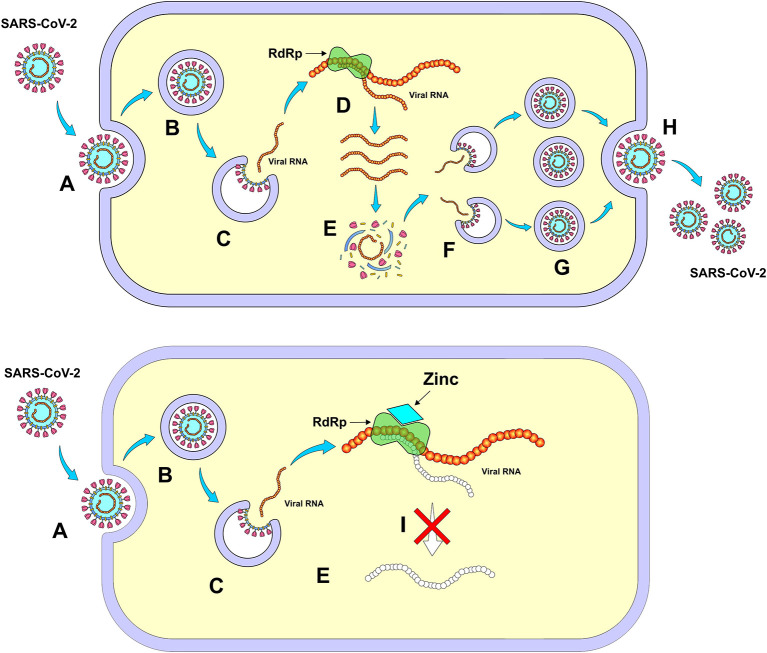
Mechanism of action of zinc – Inhibition of RNA-dependent RNA polymerase (RdRp). **(A)** Entry of SARS-CoV-2 into the cell, **(B)** Formation of endosome, **(C)** Release of viral RNA in the cell cytoplasm, **(D)** Viral self-replication by RdRp, **(E)** Synthesis of virus structural proteins, **(F)** Incorporation of RNA viral, **(G)** New viral units, **(H)** Release of new viruses, and **(I)** Inhibition of viral self-replication by RdRp. *Figure source: Authors' own drawing*.

An *in vitro* study demonstrated that the intracellular increase in Zn+2 associated with its ionophore pyrithione at concentrations of 2/2 μM, was able to inhibit viral replication of SARS-CoV and equine arteritis virus in cell cultures. According to the authors, the antiviral activity of Zn+2 is attributed to the inhibition of the RNA-dependent RNA polymerase (RdRp) responsible for the transcription of the viral genome (Figure 10) (116). Additionally, Read et al. pointed out that zinc has other properties such as a direct inhibitory activity on other viruses, as well as inhibition of the formation of the viral coating and processing of its structural components (115).

The use of 75 mg/day of zinc has been able to reduce the severity of the cases and the period of illness in patients with viral infections. It is recommended though that the use of zinc should be started within the first 24 h of the onset of the infection symptoms and that its daily administration should be maintained throughout the disease period (117). On the other hand, Zhang and Liu state in their systematic review that the association of zinc and pyrithione at low concentrations contributes to the reduction of SARS replication (SARS-CoV), therefore having a direct antiviral effect (118).

Finally, Xue et al. demonstrated a synergistic effect of chloroquine with zinc in terms of the cytotoxic effect on cancer cells, opening a new possibility of association between antimalarial and zinc for other conditions such as viral infections (119).

## Discussion

At the time of writing this review, no drug has proven to be fully effective against COVID-19, however, regulatory agencies from all over the world are cautious by only supporting the use of agents whose effectiveness has been proved under certain conditions and based on promising results from reliable studies.

Throughout this review, scientific evidence for multiple therapeutic combinations was discussed, in which the studies have shown greater efficacy in comparison to each treatment individually. For instance, the increase in the effectiveness of the treatment has been documented for the association of hydroxychloroquine and azithromycin (77) as well as for lopinavir and ritonavir (20, 51, 62). In addition, in hospitalized patients with severe acute respiratory distress syndrome, interventions are indicated to control coagulation disorders and exacerbated inflammatory response as well as to increase the immune response. Thus, in severe or life-threatening COVID-19 patients, most guidelines indicate the association of anticoagulants, corticosteroids, antibiotics and immunity mediators. Furthermore, it seems that the use of multi-therapy by associating different therapeutic agents that act through distinct mechanisms of action is a promising alternative to overcome this current health crisis until a fully effective drug or vaccine is discovered.

In some countries, the prophylaxis of COVID-19 for health professionals, elderly and infected contacts has been proposed with drugs that have shown *in vitro* antiviral activity (67), which includes chloroquine, hydroxychloroquine and ivermectin. However, randomized clinical trials have not demonstrated a prophylactic effect with reduced hospitalization among adults with mild COVID-19 that have been treated with hydroxychloroquine. In addition, there are no randomized studies that prove the efficacy and safety of ivermectin prophylaxis against SARS-CoV-2. Therefore, the existing evidences do not support that the benefits of such prophylactic treatment outweigh the risks and we advise that all the risks must be clearly explained to patients who seek protection against SARS-CoV-2 by using these drugs.

**Figure 11:**
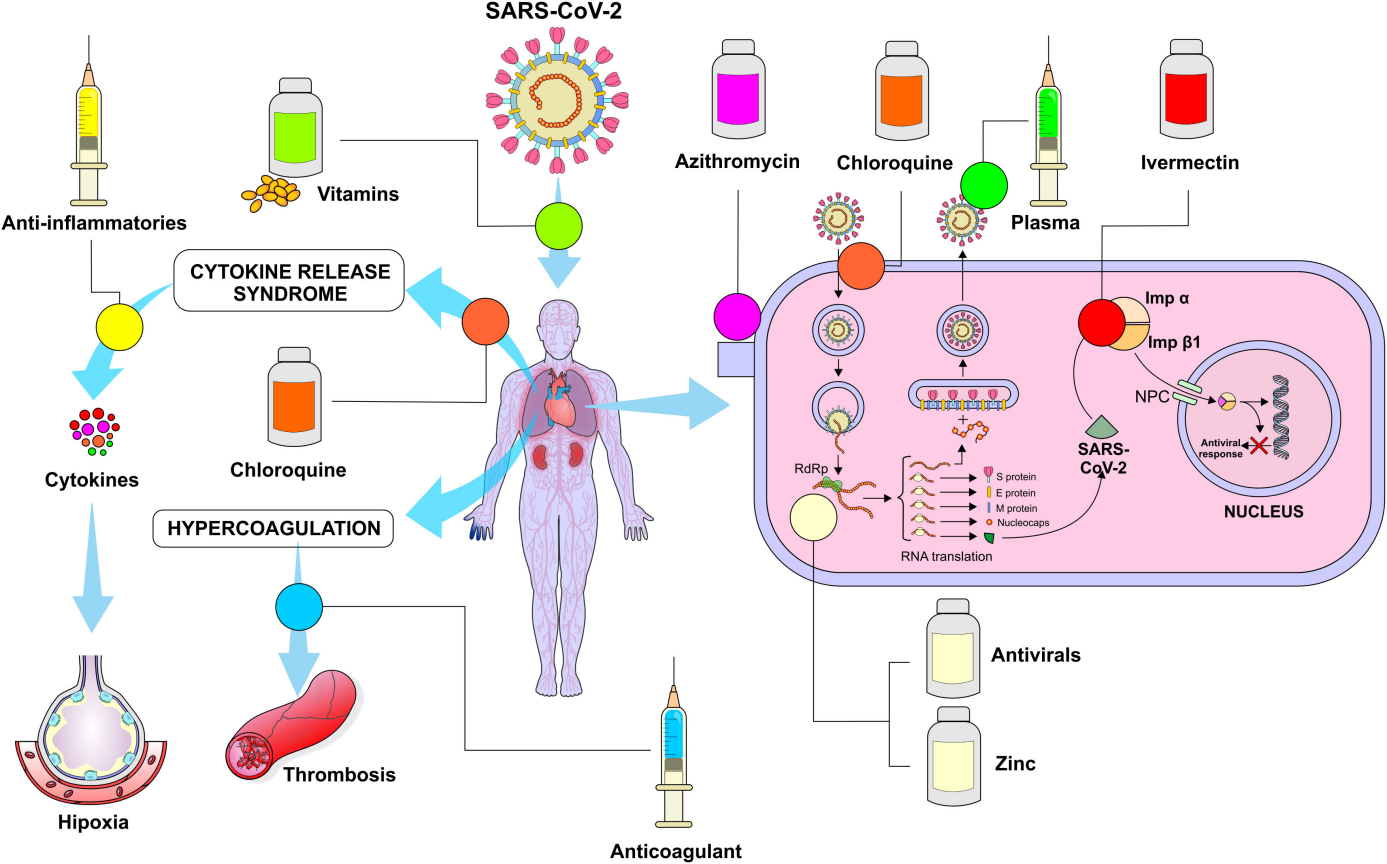
Summary of the mechanisms of action of the drugs currently used in SARS-CoV-2 infection. Chloroquine – Interferes with ACE2 ligands and receptors, decreasing the penetration of the virus into the cell, in addition, it changes the pH of the endosome, making it difficult to release viral RNA in the cell's cytoplasm; Azithromycin - Reduces the number of microorganisms in the alveolus and maintains the integrity of tight junctions, reinforcing the virus barrier; Anti-inflammatory drugs - Reduce the inflammatory process by decreasing the release of cytokines, Ivermectin - Inhibits the integrase protein and importin α/β1 (IMPα/β1) heterodimer that promote the entry of viral proteins in the cell nucleus; Convalescent plasma - Immunoglobulins directly fight the virus; Anticoagulant drugs- Interact with antithrombin reducing the thrombotic process; Antivirals (Remdesivir) - Inhibit the RNA-dependent RNA polymerase (RdRp) preventing the self-replication of viral RNA; Zinc - inhibits RNA-dependent RNA polymerase (RdRp) preventing self-replication of viral RNA. *Figure source: Authors' own drawing*.

Multiple treatment of COVID-19 has been adopted (Figure 11) from a responsible perspective, given the massive need to adopt important therapeutic measures in an increasingly intense relationship of disease severity and time. It is important to reinforce precautions regarding the side effects of some drugs and that in many cases only off-label and compassionate use are justified.

The development of more research to find a more specific drug to treat this disease has been ensured by the scientific community, preserving the bioethical principles of research involving human beings. We hope that as the several randomized clinical trials are being conducted worldwide, the drugs with the best efficacy and safety profile will soon be found.

## Author Contributions

AF wrote about antivirals, ivermectin, nitazoxanide, and organized the entire references. AV wrote about chloroquine and hydroxychloroquine. FG wrote about anti-inflammatory drugs, vitamins and helped with selecting the articles, and writing the conclusion section. FP wrote about zinc and drew all figures included in this manuscript. RC wrote about anticoagulants, convalescent plasma, organized the abstract, and methodology sections. EA wrote about azithromycin, vitamin D, and translated the entire text to English. All authors approved the final version of the manuscript before submission.

## Conflict of Interest

The authors declare that the research was conducted in the absence of any commercial or financial relationships that could be construed as a potential conflict of interest.

## References

[1] Poon LC Yang H Kapur A Melamed N Dao B Divakar H. Global interim guidance on coronavirus disease 2019 (COVID-19) during pregnancy and puerperium from FIGO and allied partners: information for healthcare professionals. Int J Gynaecol Obstet. (2020) 149:273–86. 10.1002/ijgo.13156 32248521 PMC9087575

[2] WHO Director-General's Opening Remarks at the Media Briefing on COVID-19 (2020). Available online at: https://www.who.int/dg/speeches/detail/who-director-general-s-opening-remarks-at-the-media-briefing-on-covid-19—15-june-2020 (accessed April 19, 2020).

[3] WHO Director-General's Opening Remarks at the Media Briefing on COVID-19 (2020). Available online at: https://www.who.int/dg/speeches/detail/who-director-general-s-opening-remarks-at-the-media-briefing-on-covid-19—30-march-2020 (accessed April 19, 2020).

[4] World Health Organization Coronavirus disease 2019 (COVID-19). Situation Report−163. (2020). Available online at: https://www.who.int/emergencies/diseases/novel-coronavirus-2019/situation-reports (accessed July 2, 2020).

[5] Syal K. COVID-19: herd immunity and convalescent plasma transfer therapy. J Med Virol. (2020) 92:1380–2. 10.1002/jmv.25870 32281679 PMC7262166

[6] Li H Zhou Y Zhang M Wang H Zhao Q Liu J. Updated approaches against SARS-CoV-2. Antimicrob Agents Chemother. (2020) 64:e00483-20. 10.1128/AAC.00483-20 32205349 PMC7269512

[7] Bein B Bachmann M Huggett S Wegermann P. [SARS CoV-2/COVID-19: evidence-based recommendation on diagnosis and therapy]. Anasthesiol Intensivmed Notfallmed Schmerzther. (2020) 55:257–65. 10.1055/a-1146-8674 32274773 PMC7295266

[8] McCreary EK Pogue JM. Coronavirus disease 2019 treatment: a review of early and emerging options. Open forum Infect Dis. (2020) 7:ofaa105. 10.1093/ofid/ofaa105 32284951 PMC7144823

[9] Sanders JM Monogue ML Jodlowski TZ Cutrell JB. Pharmacologic Treatments for coronavirus disease 2019 (COVID-19): a review. JAMA. (2020) 323:1824–6. 10.1001/jama.2020.6019 32282022

[10] Yao XH Li TY He ZC Ping YF Liu HW Yu SC. [A pathological report of three COVID-19 cases by minimally invasive autopsies]. Zhonghua bing li xue za zhi. (2020) 49:E009. 10.3760/cma.j.cn112151-20200312-00193 32172546

[11] Huang C Wang Y Li X Ren L Zhao J Hu Y. Clinical features of patients infected with 2019 novel coronavirus in Wuhan, China. Lancet. (2020) 395:497–506. 10.1016/S0140-6736(20)30183-5 31986264 PMC7159299

[12] Gautret P Lagier J-C Parola P Hoang VT Meddeb L Mailhe M. Hydroxychloroquine and azithromycin as a treatment of COVID-19: results of an open-label non-randomized clinical trial. Int J Antimicrob Agents. (2020) 56:105949. 10.1016/j.ijantimicag.2020.105949 32205204 PMC7102549

[13] Lagier J-C Million M Gautret P Colson P Cortaredona S Giraud-Gatineau A. Outcomes of 3,737 COVID-19 patients treated with hydroxychloroquine/azithromycin and other regimens in Marseille, France: A retrospective analysis. Travel Med Infect Dis. (2020) 36:101791. 10.1016/j.tmaid.2020.101791 32593867 PMC7315163

[14] Mitjà O Corbacho-Monné M Ubals M Tebe C Peñafiel J Tobias A. Hydroxychloroquine for Early treatment of adults with mild Covid-19: a randomized-controlled trial. Clin Infect Dis. (2020) 16:ciaa1009. 10.1093/cid/ciaa1009 32674126 PMC7454406

[15] Skipper CP Pastick KA Engen NW Bangdiwala AS Abassi M Lofgren SM. Hydroxychloroquine in nonhospitalized adults with early COVID-19. Ann Intern Med. (2020) 16:M20-4207. 10.7326/M20-4207 32673060 PMC7384270

[16] Cavalcanti AB Zampieri FG Rosa RG Azevedo LCP Veiga VC Avezum A Hydroxychloroquine with or without azithromycin in mild-to-moderate Covid-19. N Engl J Med. (2020) NEJMoa2019014 1:12. 10.1056/NEJMoa2019014 PMC7397242 32706953

[17] Borba MGS Val FFA Sampaio VS Alexandre MAA Melo GC Brito M Effect of high vs low doses of chloroquine diphosphate as adjunctive therapy for patients hospitalized with severe acute respiratory syndrome coronavirus 2 (SARS-CoV-2) infection: a randomized clinical trial. JAMA Netw Open. (2020) 3:e208857 10.1001/jamanetworkopen.2020.8857 32330277 PMC12124691

[18] Tang N Bai H Chen X Gong J Li D Sun Z Anticoagulant treatment is associated with decreased mortality in severe coronavirus disease 2019 patients with coagulopathy. J Thromb Haemost. (2020) 18:1094–9. 10.1111/jth.14817 32220112 PMC9906401

[19] Duan K Liu B Li C Zhang H Yu T Qu J. Effectiveness of convalescent plasma therapy in severe COVID-19 patients. Proc Natl Acad Sci USA. (2020) 117:9490–6. 10.1073/pnas.2007408117 32253318 PMC7196837

[20] HAN Archive - 00429 | Health Alert Network (HAN) (2020). Available online at: https://emergency.cdc.gov/han/2020/han00429.asp (accessed April 19, 2020).

[21] Wang Y Fan G Salam A Horby P Hayden FG Chen C. Comparative effectiveness of combined favipiravir and oseltamivir therapy versus oseltamivir monotherapy in critically Ill patients with influenza virus infection. J Infect Dis. (2019) 221:1688–98. 10.1093/infdis/jiz656 31822885

[22] Goldman JD Lye DCB Hui DS Marks KM Bruno R Montejano R Remdesivir for 5 or 10 days in patients with severe Covid-19. N Engl J Med. (2020) NEJMoa2015301:1–11. 10.1056/NEJMoa2015301. [Epub ahead of print]. PMC7377062 32459919

[23] Wang Y Zhang D Du G Du R Zhao J Jin Y. Remdesivir in adults with severe COVID-19: a randomised, double-blind, placebo-controlled, multicentre trial. Lancet. (2020) 395:1569–78. 10.1056/NEJMoa2007764 32423584 PMC7190303

[24] Chen N Zhou M Dong X Qu J Gong F Han Y. Epidemiological and clinical characteristics of 99 cases of 2019 novel coronavirus pneumonia in Wuhan, China: a descriptive study. Lancet. (2020) 395:507–13. 10.1016/S0140-6736(20)30211-7 32007143 PMC7135076

[25] Caly L Druce JD Catton MG Jans DA Wagstaff KM. The FDA-approved drug ivermectin inhibits the replication of SARS-CoV-2 in vitro. Antiviral Res. (2020) 178:104787. 10.1016/j.antiviral.2020.104787 32251768 PMC7129059

[26] Rossignol JF. Nitazoxanide, a new drug candidate for the treatment of Middle East respiratory syndrome coronavirus. J Infect Public Health. (2016) 9:227–30. 10.1016/j.jiph.2016.04.001 27095301 PMC7102735

[27] Grant WB Lahore H McDonnell SL Baggerly CA French CB Aliano JL Evidence that vitamin D Supplementation could reduce risk of influenza and COVID-19 infections and deaths. (2020) 12:988 10.20944/preprints202003.0235.v2 PMC7231123 32252338

[28] Yin S Huang M Li D Tang N. Difference of coagulation features between severe pneumonia induced by SARS-CoV2 and non-SARS-CoV2. J Thromb Thrombolysis. (2020) 3:1–4. 10.1007/s11239-020-02105-8 32246317 PMC7124128

[29] Li H Liu L Zhang D. SARS-CoV-2 and viral sepsis: observations and hypotheses. Lancet. (2020) 395:1517–20. 10.1016/S0140-6736(20)30920-X 32311318 PMC7164875

[30] Ozolina A Sarkele M Sabelnikovs O Skesters A Jaunalksne I Serova J. Activation of coagulation and fibrinolysis in acute respiratory distress syndrome: A prospective pilot study. Front Med. (2016) 3:64. 10.3389/fmed.2016.00064 27965960 PMC5125303

[31] Connors JM Levy JH. COVID-19 and its implications for thrombosis and anticoagulation. Blood. (2020) 135:2033–40. 10.1182/blood.2020006000 32339221 PMC7273827

[32] Li J Li Y Yang B Wang H Li L Low-molecular-weight heparin treatment for acute lung injury/acute respiratory distress syndrome: a meta-analysis of randomized controlled trials. Int J Clin Exp Med. (2018) 11:414–22.

[33] Seffer M-T Cottam D Forni LG Kielstein JT. Heparin 2.0: a new approach to the infection crisis. Blood Purif. (2020) 1:1–7. 10.1159/000508647 32615569 PMC7445380

[34] Thachil J Tang N Gando S Falanga A Cattaneo M Levi M. ISTH interim guidance on recognition and management of coagulopathy in COVID-19. J Thromb Haemost. (2020) 18:1023–26. 10.1111/jth.14810 32338827 PMC9906133

[35] Gao Y Li T Han M Li X Wu D Xu Y. Diagnostic utility of clinical laboratory data determinations for patients with the severe COVID-19. J Med Virol. (2020) 92:791–6. 10.1002/jmv.25770 32181911 PMC7228247

[36] Chen C Zhang XR Ju ZY He WF [Advances in the research of cytokine storm mechanism induced by corona virus disease 2019 and the corresponding immunotherapies]. Zhonghua Shao Shang Za Zhi. (2020) 36:E005 10.3760/cma.j.cn501120-20200224-00088 32114747

[37] Qin C Zhou L Hu Z Zhang S Yang S Tao Y. Dysregulation of immune response in patients with COVID-19 in Wuhan, China. Clin Infect Dis. (2020) 71:762–8. 10.1093/cid/ciaa248 32161940 PMC7108125

[38] Wu C Chen X Cai Y Xia J Zhou X Xu S. Risk factors associated with acute respiratory distress syndrome and death in patients with coronavirus disease 2019 pneumonia in Wuhan, China. JAMA Intern Med. (2020) 180:1–11. 10.1001/jamainternmed.2020.0994 32167524 PMC7070509

[39] Conti P Ronconi G Caraffa A Gallenga CE Ross R Frydas I. Induction of pro-inflammatory cytokines (IL-1 and IL-6) and lung inflammation by Coronavirus-19 (COVI-19 or SARS-CoV-2): anti-inflammatory strategies. J Biol Regul Homeost Agents. (2020) 34:327–31. 10.23812/CONTI-E 32171193

[40] Ruan Q Yang K Wang W Jiang L Song J Clinical predictors of mortality due to COVID-19 based on an analysis of data of 150 patients from Wuhan, China. Intensive Care Med. (2020) 46:846–8. 10.1007/s00134-020-05991-x 32125452 PMC7080116

[41] Zhang W Zhao Y Zhang F Wang Q Li T Liu Z. The use of anti-inflammatory drugs in the treatment of people with severe coronavirus disease 2019 (COVID-19): The experience of clinical immunologists from China. Clin Immunol. (2020) 214:108393. 10.1016/j.clim.2020.108393 32222466 PMC7102614

[42] Wang D Hu B Hu C Zhu F Liu X Zhang J. Clinical characteristics of 138 hospitalized patients with 2019 novel coronavirus-infected pneumonia in Wuhan, China. JAMA - J Am Med Assoc. (2020) 323:1061–9. 10.1001/jama.2020.1585 32031570 PMC7042881

[43] Auyeung TW Lee JSW Lai WK Choi CH Lee HK Lee JS. The use of corticosteroid as treatment in SARS was associated with adverse outcomes: a retrospective cohort study. J Infect. (2005) 51:98–102. 10.1016/j.jinf.2004.09.008 16038758 PMC7132384

[44] Jin YH Cai L Cheng ZS Cheng H Deng T Fan YP A rapid advice guideline for the diagnosis and treatment of 2019 novel coronavirus (2019-nCoV) infected pneumonia (standard version). Mil Med Res. (2020) 7:4 10.1186/s40779-020-0233-6 32029004 PMC7003341

[45] Russell CD Millar JE Baillie JK. Clinical evidence does not support corticosteroid treatment for 2019-nCoV lung injury. Lancet. (2020) 395:473–5. 10.1016/S0140-6736(20)30317-2 32043983 PMC7134694

[46] Arabi YM Mandourah Y Al-Hameed F Sindi AA Almekhlafi GA Hussein MA. Corticosteroid therapy for critically ill patients with middle east respiratory syndrome. Am J Respir Crit Care Med. (2018) 197:757–67. 10.1164/rccm.201706-1172OC 29161116

[47] Griffith JF Antonio GE Kumta SM Hui DSC Wong JKT Joynt GM. Osteonecrosis of hip and knee in patients with severe acute respiratory syndrome treated with steroids. Radiology. (2005) 235:168–75. 10.1148/radiol.2351040100 15703312

[48] Lee N Allen Chan KC Hui DS Ng EKO Wu A Chiu RWK. Effects of early corticosteroid treatment on plasma SARS-associated Coronavirus RNA concentrations in adult patients. J Clin Virol. (2004) 31:304–9. 10.1016/j.jcv.2004.07.006 15494274 PMC7108318

[49] Ling Y Xu S-B Lin Y-X Tian D Zhu Z-Q Dai F-H. Persistence and clearance of viral RNA in 2019 novel coronavirus disease rehabilitation patients. Chin Med J. (2020) 133:1039–43 10.1097/CM9.0000000000000774 32118639 PMC7147278

[50] Siddiqi HK Mehra MR. COVID-19 illness in native and immunosuppressed states: a clinical-therapeutic staging proposal. J Hear Lung Transplant. (2020) 39:405–7 10.1016/j.healun.2020.03.012 32362390 PMC7118652

[51] World Health Organization Clinical Management of Severe Acute Respiratory Infection When Novel Coronavirus (2019-nCoV) Infection is Suspected: Interim Guidance. Geneva: World Health Organization (2020). Available online at: https://extranet.who.int/iris/restricted/handle/10665/330893

[52] Richardson P Griffin I Tucker C Smith D Oechsle O Phelan A. Baricitinib as potential treatment for 2019-nCoV acute respiratory disease. Lancet. (2020) 395:e30–1. 10.1016/S0140-6736(20)30304-4 32032529 PMC7137985

[53] Lewis SR Pritchard MW Thomas CM Smith AF. Pharmacological agents for adults with acute respiratory distress syndrome. Cochrane Database Syst Rev. (2019) 2019:CD004477. 10.1002/14651858.CD004477.pub3 31334568 PMC6646953

[54] Shang L Zhao J Hu Y Du R Cao B On the use of corticosteroids for 2019-nCoV pneumonia. Lancet. (2020) 395:683–4. 10.1016/S0140-6736(20)30361-5 32122468 PMC7159292

[55] Horby P Lim WS Emberson J Mafham M Bell J Linsell L. Effect of dexamethasone in hospitalized patients with COVID-19: preliminary report. medRxiv. (2020) 1–11. 10.1101/2020.06.22.2013727332678530

[56] Callejas Rubio JL Luna del Castillo J de D de la Hera Fernández J Guirao Arrabal E Colmenero Ruiz M Ortego Centeno N Effectiveness of corticoid pulses in patients with cytokine storm syndrome induced by SARS-CoV-2 infection. Med Clin. (2020) 155:159–61. 10.1016/j.medcle.2020.07.002 PMC7351423 32835105

[57] Selvaraj V Dapaah-Afriyie K Finn A Flanigan T. Short-term corticosteroids in SARS-CoV2 patients: hospitalists' perspective. medRxiv. (2020). 10.1101/2020.06.19.2010917332570995

[58] Fadel R Morrison AR Vahia A Smith ZR Chaudhry Z Bhargava P. Early short course corticosteroids in hospitalized patients with COVID-19. Clin Infect Dis. (2020) 19:ciaa601. 10.1101/2020.05.04.20074609 32427279 PMC7314133

[59] Keller MJ Kitsis EA Arora S Chen J-T Agarwal S Ross MJ Effect of systemic glucocorticoids on mortality or mechanical ventilation in patients with COVID-19. J Hosp Med. (2020) 22:2020 10.12788/jhm.3497 PMC7518134 32804611

[60] Lu H. Drug treatment options for the 2019-new coronavirus (2019-nCoV). Biosci Trends. (2020) 14:69–71. 10.5582/bst.2020.01020 31996494

[61] World Health Organization Novel Coronavirus (2019-nCoV) SITUATION REPORT - 1. (2020). Available online at: https://www.who.int/docs/default-source/coronaviruse/situation-reports/20200121-sitrep-1-2019-ncov.pdf (accessed July 25, 2020).

[62] Li H Wang YM Xu JY Cao B [Potential antiviral therapeutics for 2019 novel coronavirus]. Zhonghua Jie He He Hu Xi Za Zhi. (2020) 43:170–2. 10.3760/cma.j.issn.1001-0939.2020.03.004 32164080

[63] Cao Y Deng Q Dai S. Remdesivir for severe acute respiratory syndrome coronavirus 2 causing COVID-19: An evaluation of the evidence. Travel Med Infect Dis. (2020) 35:101647. 10.1016/j.tmaid.2020.101647 32247927 PMC7151266

[64] Elfiky AA. Ribavirin, remdesivir, sofosbuvir, galidesivir, and tenofovir against SARS-CoV-2 RNA dependent RNA polymerase (RdRp): a molecular docking study. Life Sci. (2020) 253:117592. 10.1016/j.lfs.2020.117592 32222463 PMC7102646

[65] Jefferson T Jones MA Doshi P Del Mar CB Hama R Thompson MJ. Neuraminidase inhibitors for preventing and treating influenza in healthy adults and children. Cochrane database Syst Rev. (2014) 2014:CD008965. 10.1002/14651858.CD008965.pub4 22258996

[66] Yang SNY Atkinson SC Wang C Lee A Bogoyevitch MA Borg NA. The broad spectrum antiviral ivermectin targets the host nuclear transport importin α/β1 heterodimer. Antiviral Res. (2020) 177:104760. 10.1016/j.antiviral.2020.104760 32135219

[67] Gupta D Sahoo AK Singh A. Ivermectin: potential candidate for the treatment of Covid 19. Br J Infect Dis. (2020). 10.1016/j.bjid.2020.06.002 32615072 PMC7321032

[68] Heidary F Gharebaghi R. Ivermectin: a systematic review from antiviral effects to COVID-19 complementary regimen. J Antibiot. (2020) 73:593–602. 10.1038/s41429-020-0336-z 32533071 PMC7290143

[69] Srivatsan P Potential dual therapeutic approach against SARS-CoV-2/COVID-19 with nitazoxanide and hydroxychloroquine. Preprint. (2020).

[70] Teoh K-T Siu Y-L Chan W-L Schlüter MA Liu C-J Peiris JSM. The SARS coronavirus E protein interacts with PALS1 and alters tight junction formation and epithelial morphogenesis. Mol Biol Cell. (2010) 21:3838–52. 10.1091/mbc.e10-04-0338 20861307 PMC2982091

[71] Asgrimsson V Gudjonsson T Gudmundsson GH Baldursson O. Novel effects of azithromycin on tight junction proteins in human airway epithelia. Antimicrob Agents Chemother. (2006) 50:1805–12. 10.1128/AAC.50.5.1805-1812.2006 16641453 PMC1472195

[72] Min JY Jang YJ. Macrolide therapy in respiratory viral infections. Mediat Inflamm. (2012) 2012:649570. 10.1155/2012/649570 22719178 PMC3375106

[73] Tran DH Sugamata R Hirose T Suzuki S Noguchi Y Sugawara A. Azithromycin, a 15-membered macrolide antibiotic, inhibits influenza A(H1N1)pdm09 virus infection by interfering with virus internalization process. J Antibiot. (2019) 72:759–68. 10.1038/s41429-019-0204-x 31300721

[74] Bosseboeuf E Aubry M Nhan T de Pina JJ Rolain JM Raoult D Azithromycin inhibits the replication of Zika virus. J Antivir Antiretrovir. (2018) 10:6–11. 10.4172/1948-5964.1000173

[75] Bixler SL Duplantier AJ Bavari S. Discovering Drugs for the treatment of ebola virus. Curr Treat Options Infect Dis. (2017) 9:299–317. 10.1007/s40506-017-0130-z 28890666 PMC5570806

[76] Gabriels J Saleh M Chang D Epstein LM. Inpatient Use of mobile continuous telemetry for COVID-19 patients treated with hydroxychloroquine and azithromycin. Hear Case Reports. (2020) 6:241–3. 10.1016/j.hrcr.2020.03.017 32363144 PMC7194904

[77] Arshad S Kilgore P Chaudhry ZS Jacobsen G Wang DD Huitsing K. Treatment with hydroxychloroquine, azithromycin, and combination in patients hospitalized with COVID-19. Int J Infect Dis. (2020) 97:396–403. 10.1016/j.ijid.2020.06.099 32623082 PMC7330574

[78] Yao X Ye F Zhang M Cui C Huang B Niu P. *In vitro* antiviral activity and projection of optimized dosing design of hydroxychloroquine for the treatment of severe acute respiratory syndrome coronavirus 2 (SARS-CoV-2). Clin Infect Dis. (2020) 71:732–9. 10.1093/cid/ciaa237 32150618 PMC7108130

[79] Hong W. Combating COVID-19 with chloroquine. J Mol Cell Biol. (2020) 12:249–50. 10.1093/jmcb/mjaa015 32236561 PMC7184415

[80] Pacheco R Pachito D Bagattini A Riera R Hidroxicloroquina e cloroquina para infecção por COVID-19 (2020). Available online at: https://oxfordbrazilebm.com/wp-content/uploads/2020/03/RS_rapida_hidroxi_cloroquina_COVID19.pdf (accessed August 06, 2020).

[81] Gao J Tian Z Yang X. Breakthrough: Chloroquine phosphate has shown apparent efficacy in treatment of COVID-19 associated pneumonia in clinical studies. Biosci Trends. (2020) 14:72–3. 10.5582/bst.2020.01047 32074550

[82] Monteiro WM Brito-Sousa JD Baía-da-Silva D Melo GC de Siqueira AM Val F. Driving forces for COVID-19 clinical trials using chloroquine: the need to choose the right research questions and outcomes. Rev Soc Bras Med Trop. (2020) 53:e20200155. 10.1590/0037-8682-0155-2020 32267301 PMC7156252

[83] Tobón C Palacio LC Chidipi B Slough DP Tran T Tran N. The antimalarial chloroquine reduces the burden of persistent atrial fibrillation. Front Pharmacol. (2019) 10:1392. 10.3389/fphar.2019.01392 31827438 PMC6890839

[84] Rodney R 2019 Novel Coronavirus (2019-nCoV) Update: Uncoating the Virus. Am Soc Microbiol. (2020).

[85] Coronavirus disease 2019 (COVID-19): Epidemiology Virology Clinical Features Diagnosis and Prevention (2020). Available online at: https://www.uptodate.com/contents/coronavirus-disease-2019-covid-19-epidemiology-virology-clinical-features-diagnosis-and-prevention (accessed April 19, 2020).

[86] McIntosh K Hirsh MS Bloom A Coronavirus Disease 2019 (COVID-19): Epidemiology, Virology, Clinical Features, Diagnosis, and Prevention. (2020). Available online at: https://www.uptodate.com/contents/coronavirus-disease-2019-covid-19-epidemiology-virology-clinical-features-diagnosis-and-prevention (accessed April 19, 2020).

[87] Colson P Rolain JM Lagier JC Brouqui P Raoult D. Chloroquine and hydroxychloroquine as available weapons to fight COVID-19. Int J Antimicrob Agents. (2020) 55:105932. 10.1016/j.ijantimicag.2020.105932 32145363 PMC7135139

[88] Chorin E Dai M Shulman E Wadhwani L Bar-Cohen R Barbhaiya C. The QT interval in patients with COVID-19 treated with hydroxychloroquine and azithromycin. Nat Med. (2020) 26:808–809. 10.1038/s41591-020-0888-2 32488217

[89] Zhou D Dai S-M Tong Q. COVID-19: a recommendation to examine the effect of hydroxychloroquine in preventing infection and progression. J Antimicrob Chemother. (2020) 75:1667–70. 10.1093/jac/dkaa114 32196083 PMC7184499

[90] Garraud O Heshmati F Pozzetto B Lefrere F Girot R Saillol A. Plasma therapy against infectious pathogens, as of yesterday, today and tomorrow. Transfus Clin Biol. (2016) 23:39–44. 10.1016/j.tracli.2015.12.003 26775794 PMC7110444

[91] Sahr F Ansumana R Massaquoi TA Idriss BR Sesay FR Lamin JM. Evaluation of convalescent whole blood for treating ebola virus disease in freetown, sierra leone. J Infect. (2017) 74:302–9. 10.1016/j.jinf.2016.11.009 27867062 PMC7112610

[92] Mair-Jenkins J Saavedra-Campos M Baillie JK Cleary P Khaw F-M Lim WS. The effectiveness of convalescent plasma and hyperimmune immunoglobulin for the treatment of severe acute respiratory infections of viral etiology: a systematic review and exploratory meta-analysis. J Infect Dis. (2014) 211:80–90. 10.1093/infdis/jiu396 25030060 PMC4264590

[93] Shen C Wang Z Zhao F Yang Y Li J Yuan J. Treatment of 5 critically Ill patients with COVID-19 with convalescent plasma. JAMA - J Am Med Assoc. (2020) 323:1582–9. 10.1001/jama.2020.4783 32219428 PMC7101507

[94] Roback JD Guarner J. Convalescent plasma to treat COVID-19: possibilities and challenges. JAMA - J Am Med Assoc. (2020) 16:1561–62. 10.1001/jama.2020.4940 32219429

[95] Valk SJ Piechotta V Chai KL Doree C Monsef I Wood EM Convalescent plasma or hyperimmune immunoglobulin for people with COVID-19: a rapid review. Cochrane Database Syst Rev. (2020) 5:CD013600 10.1002/14651858.CD013600 32406927 PMC7271896

[96] Li L Zhang W Hu Y Tong X Zheng S Yang J. Effect of convalescent plasma therapy on time to clinical improvement in patients with severe and life-threatening COVID-19: A randomized clinical trial. JAMA. (2020) 324:1–11. 10.1001/jama.2020.12607 32492084 PMC7270883

[97] Spyropoulos AC Ageno W Barnathan ES. Hospital-based use of thromboprophylaxis in patients with COVID-19. Lancet. (2020) 395:e75. 10.1016/S0140-6736(20)30926-0 32330428 PMC7173816

[98] Bikdeli B Madhavan M V Jimenez D Chuich T Dreyfus I Driggin E. COVID-19 and thrombotic or thromboembolic disease: implications for prevention, antithrombotic therapy, and follow-up: JACC State-of-the-art review. J Am Coll Cardiol. (2020) 75:2950–73. 10.1016/j.jacc.2020.04.031 32311448 PMC7164881

[99] Beard JA Bearden A Striker R. Vitamin D and the anti-viral state. J Clin Virol. (2011) 50:194–200. 10.1016/j.jcv.2010.12.006 21242105 PMC3308600

[100] Kandeel M Al-Nazawi M. Virtual screening and repurposing of FDA approved drugs against COVID-19 main protease. Life Sci. (2020) 251:117627. 10.1016/j.lfs.2020.117627 32251634 PMC7194560

[101] Hemilä H. Vitamin C intake and susceptibility to pneumonia. Pediatr Infect Dis J. (1997) 16:836–7. 10.1097/00006454-199709000-00003 9306475

[102] Nonnecke BJ McGill JL Ridpath JF Sacco RE Lippolis JD Reinhardt TA. Acute phase response elicited by experimental bovine diarrhea virus (BVDV) infection is associated with decreased vitamin D and E status of vitamin-replete preruminant calves. J Dairy Sci. (2014) 97:5566–79. 10.3168/jds.2014-8293 25022687

[103] Lippi G Henry BM Bovo C Sanchis-Gomar F. Health risks and potential remedies during prolonged lockdowns for coronavirus disease 2019 (COVID-19). Diagnosis. (2020) 7:85–90. 10.1515/dx-2020-0041 32267243

[104] Teymoori-Rad M Shokri F Salimi V Marashi SM. The interplay between vitamin D and viral infections. Rev Med Virol. (2019) 29:e2032. 10.1002/rmv.2032 30614127

[105] Jiménez-Sousa M ángeles Martínez I Medrano LM Fernández-Rodríguez A Resino S. Vitamin D in human immunodeficiency virus infection: influence on immunity and disease. Front Immunol. (2018) 9:458. 10.3389/fimmu.2018.00458 29593721 PMC5857570

[106] Havers F Smeaton L Gupte N Detrick B Bollinger RC Hakim J. 25-Hydroxyvitamin D insufficiency and deficiency is associated with HIV disease progression and virological failure post-antiretroviral therapy initiation in diverse multinational settings. J Infect Dis. (2014) 210:244–53. 10.1093/infdis/jiu259 24799602 PMC4141201

[107] Gruber-Bzura BM Vitamin D and influenza—Prevention or therapy? Int J Mol Sci. (2018) 19:2419 10.3390/ijms19082419 PMC6121423 30115864

[108] Searing DA Zhang Y Murphy JR Hauk PJ Goleva E Leung DYM. Decreased serum vitamin D levels in children with asthma are associated with increased corticosteroid use. J Allergy Clin Immunol. (2010) 125:995–1000. 10.1016/j.jaci.2010.03.008 20381849 PMC2866800

[109] Martineau AR Jolliffe DA Hooper RL Greenberg L Aloia JF Bergman P. Vitamin D supplementation to prevent acute respiratory tract infections: systematic review and meta-analysis of individual participant data. BMJ. (2017) 356:i6583. 10.1136/bmj.i6583 28202713 PMC5310969

[110] Muscogiuri G Barrea L Savastano S Colao A. Nutritional recommendations for CoVID-19 quarantine. Eur J Clin Nutr. (2020) 74:850–1. 10.1038/s41430-020-0635-2 32286533 PMC7155155

[111] Watkins J. Preventing a covid-19 pandemic. BMJ. (2020) 368:m810. 10.1136/bmj.m810 32111649

[112] Fabbri A Infante M Ricordi C. Editorial – vitamin D status: a key modulator of innate immunity and natural defense from acute viral respiratory infections. Eur Rev Med Pharmacol Sci. (2020) 24:4048–52. 10.26355/eurrev_202004_20876 32329882

[113] Maares M Haase H. Zinc and immunity: an essential interrelation. Arch Biochem Biophys. (2016) 611:58–65. 10.1016/j.abb.2016.03.022 27021581

[114] Valeska S De B Silva F Morgane M Souza1 F-RC Raque De Santana J Child zinc brain. Am J Clin Nutr. (2020) 2:123–35. 10.1093/ajcn/68.2.464S

[115] Read SA Obeid S Ahlenstiel C Ahlenstiel G. The Role of zinc in antiviral immunity. Adv Nutr. (2019) 10:696–710. 10.1093/advances/nmz013 31305906 PMC6628855

[116] te Velthuis AJW van den Worm SHE Sims AC Baric RS Snijder EJ van Hemert MJ. Zn2+ inhibits coronavirus and arterivirus RNA Polymerase activity *in vitro* and zinc ionophores block the replication of these viruses in cell culture. PLoS Pathog. (2010) 6:e1001176. 10.1371/journal.ppat.1001176 21079686 PMC2973827

[117] Singh M Das RR. Zinc for the common cold. Cochrane Database Syst Rev. (2013) 2013:CD001364. 10.1002/14651858.CD001364.pub4 23775705

[118] Zhang L Liu Y. Potential interventions for novel coronavirus in China: a systematic review. J Med Virol. (2020) 92:479–90. 10.1002/jmv.25707 32052466 PMC7166986

[119] Xue J Moyer A Peng B Wu J Hannafon BN Ding WQ. Chloroquine is a zinc ionophore. PLoS ONE. (2014) 9:e109180. 10.1371/journal.pone.0109180 25271834 PMC4182877

